# Psychometric framework for coexistence with large carnivores

**DOI:** 10.1111/cobi.70326

**Published:** 2026-05-14

**Authors:** Benjamin Ghasemi, Gerard Kyle, Tara Teel, Rebeca Niemiec, Kevin Crooks, Jenny Anne Glikman, Neil H. Carter, John A. Vucetich, Michelle L. Lute, Stewart Breck, Beatrice Frank, Dana Hoag, Jonathan Salerno, Lily M. van Eeden, Jeremy T. Bruskotter

**Affiliations:** ^1^ Department of Rangeland, Wildlife, and Fisheries Management Texas A&M University College Station Texas USA; ^2^ Department of Human Dimensions of Natural Resources Colorado State University Fort Collins Colorado USA; ^3^ Department of Fish, Wildlife, and Conservation Biology Colorado State University Fort Collins Colorado USA; ^4^ Instituto de Estudios Sociales Avanzados (IESA‐CSIC) Córdoba Spain; ^5^ School for Environment and Sustainability University of Michigan Ann Arbor Michigan USA; ^6^ College of Forest Resources and Environmental Science Michigan Technological University Houghton Michigan USA; ^7^ Wildlife for All Pena Blanca New Mexico USA; ^8^ Georgia Strait Alliance and School of Environmental Studies University of Victoria Victoria British Columbia Canada; ^9^ Department of Agricultural and Resource Economics Colorado State University Fort Collins Colorado USA; ^10^ Graduate Degree Program in Ecology Colorado State University Fort Collins Colorado USA; ^11^ School of Science RMIT University Melbourne Victoria Australia; ^12^ Department of Community Sustainability Michigan State University East Lansing Michigan USA

**Keywords:** attitudes, coexistence, human dimensions, human–wildlife conflict, intentions, predators, psychometrics, tolerance, Actitudes, coexistencia, conflicto humanos – fauna silvestre, depredadores, dimensiones humanas, intenciones, psicometría, tolerancia, 人兽冲突, 人类维度, 态度, 意图, 捕食者, 心理测量学, 共存, 容忍度

## Abstract

Human–carnivore coexistence is essential for biodiversity conservation, yet measuring the attitudes and behaviors that support it remains methodologically challenging. We developed psychometric scales to assess willingness to coexist with carnivores and the underlying beliefs of this coexistence through an iterative expert elicitation process grounded in risk perception theory and moral psychology to ensure strong content validity. The scales measure willingness to coexist and encompass individual and collective behaviors and their cognitive antecedents: perceived positive consequences, perceived negative consequences, and ethical considerations. We validated the scales based on three representative samples of Colorado residents (*n* = 434, 442, 433) focused on gray wolves (*Canis lupus*), mountain lions (*Puma concolor*), and black bears (*Ursus americanus*). Validation involved expert review, cognitive interviews, confirmatory factor analyses, and measurement invariance testing across species. The scales demonstrated strong construct validity, with factor structures consistent with theoretical expectations and evidence of convergent and discriminant validity. Concurrent validity was supported through associations with related attitudes and behavioral intentions. These instruments offer researchers a robust, theory‐driven framework for examining human–wildlife interactions that facilitates standardized comparisons across species and contexts. For practitioners, the psychometric scales provide a practical means to assess public readiness for coexistence, monitor attitudinal change over time, and design interventions tailored to specific belief structures.

## Abstract (es)

Marco psicométrico para la coexistencia con carnívoros mayores

**Resumen:**

La coexistencia entre los seres humanos y los carnívoros es esencial para la conservación de la biodiversidad; sin embargo, medir las actitudes y los comportamientos que la sustentan sigue siendo un reto desde el punto de vista metodológico. Hemos desarrollado escalas psicométricas para evaluar la disposición a coexistir con los carnívoros y las creencias subyacentes a dicha coexistencia mediante un proceso iterativo de consulta a expertos, basado en la teoría de la percepción del riesgo y la psicología moral, con el fin de garantizar una sólida validez de contenido. Las escalas miden la disposición a coexistir y abarcan comportamientos individuales y colectivos, así como sus antecedentes cognitivos: consecuencias positivas percibidas, consecuencias negativas percibidas y consideraciones éticas. Validamos las escalas basándonos en tres muestras representativas de residentes de Colorado (*n* = 434, 442, 433) centradas en los lobos grises (*Canis lupus*), los pumas (*Puma concolor*) y los osos negros (*Ursus americanus*). La validación incluyó revisiones de expertos, entrevistas cognitivas, análisis factoriales confirmatorios y pruebas de invarianza de la medición entre especies. Las escalas demostraron una sólida validez de constructo, con estructuras factoriales coherentes con las expectativas teóricas y evidencia de validez convergente y discriminante. La validez concurrente se respaldó mediante asociaciones con actitudes e intenciones de comportamiento relacionadas. Estos instrumentos ofrecen a los investigadores un marco robusto y basado en la teoría para examinar las interacciones entre los seres humanos y la fauna silvestre, lo que facilita comparaciones estandarizadas entre especies y contextos. Para los profesionales, las escalas psicométricas proporcionan un medio práctico para evaluar la disposición del público a la coexistencia, supervisar el cambio de actitud a lo largo del tiempo y diseñar intervenciones adaptadas a estructuras de creencias específicas.

## 人与大型食肉动物共存的心理测量框架

**【摘要】** 人类与食肉动物的共存对生物多样性保护至关重要, 但对支持这种共存的态度和行为开展测量评估, 仍存在方法上的挑战。本研究开发了评估与食肉动物共存的意愿及其背后信念的心理测量量表, 研究方法基于风险感知理论和道德心理学, 并通过迭代的专家征询过程, 以确保其具有强有力的内容效度。该量表用于测量共存意愿, 涵盖个体与集体行为及其认知先决条件, 包括人们感知到的积极结果、感知到的消极结果, 以及伦理考量。我们利用美国科罗拉多州居民的三组代表性样本(*n* = 434, 442, 433), 分别聚焦灰狼(*Canis lupus*)、美洲狮(*Puma concolor*)和黑熊(*Ursus americanus*), 对量表进行了验证。验证过程包括专家评审、认知访谈、确认性因子分析, 以及跨物种的测量不变性检验。我们的量表展现出很强的构念效度, 其因子结构符合理论预期, 并得到聚合效度和区分效度的证据支持。我们通过与相关态度及行为意图的关联性, 证实了其并行效度。这些工具为研究者提供了一个理论驱动的稳健框架, 可用于探究人与野生动物的互动, 并有助于在不同物种和情境间进行标准化比较。对于保护工作者而言, 这些心理测量量表提供了一种实用手段, 以评估公众对共存的准备程度、监测随时间推移的态度变化, 并设计针对特定信念结构的干预措施。**【翻译:胡怡思;审校:聂永刚】**

## INTRODUCTION

Conservation scholars increasingly recognize the importance of robust social science methods for understanding human interactions with biodiversity (e.g., Fox et al., 2006; Moon & Blackman, 2014; Moon et al., 2019). However, despite growing attention to human–carnivore coexistence as a conservation goal, there is no validated scale with which to measure this construct. As a result, conservation researchers have used a range of inconsistent and often poorly defined measures, leading to conceptual confusion, difficulties in comparing findings across studies, and limitations in applying results to conservation practice (Whitehouse‐Tedd et al., 2021). In psychology, researchers often work within a positivist framework to study unobservable latent constructs, such as attitudes, beliefs, and perceptions, by developing standardized tools for reliable measurement (Raykov & Marcoulides, 2011). A lack of standardization undermines efforts to understand and influence the human dimensions of wildlife conservation (Fox et al., 2006; Fried & Flake, 2018; Kyle et al., 2020; Teel et al., 2018).

Although structured questionnaires are commonly used in conservation psychology, Whitehouse‐Tedd et al. (2021) found that many existing measures of attitudes and tolerance toward wildlife lack rigorous psychometric validation. This limits their reliability and reduces their ability to inform effective conservation interventions. Validated psychological scales, by contrast, enhance the comparability of results across contexts, allow researchers to track changes over time, and improve the predictive accuracy of behavioral models (Breedvelt et al., 2020; Fried & Flake, 2018). For example, Carlson et al. (2023) demonstrated that theoretically distinct measures of tolerance, such as attitudes and acceptance, can have different predictors, underscoring the risks of treating these constructs interchangeably. To advance the science and practice of human–wildlife coexistence, conservation research must adopt well‐established psychometric standards, starting with the development of standardized, validated scales (Kyle et al., 2020).

To address this paucity in conservation psychology literature, we developed the willingness‐to‐coexist (WCoEx) scale to measure individuals’ willingness to coexist with carnivores. By enabling standardized measurement, the WCoEx scale could facilitate meaningful comparisons between conservation cases and support monitoring of coexistence efforts. The scale advances conservation psychology by synthesizing psychological research and coexistence theories while providing local decision makers with a practical tool to assess the social viability of human–carnivore coexistence in their regions. The scale also helps address Target 4 in the Kunming–Montreal Global Biodiversity Framework, which recognizes the need to minimize human–wildlife conflict for coexistence (Carter & Di Minin, 2024; Pooley, 2025). We conceptualized willingness to coexist (individual and collective behaviors) and its underlying beliefs (perceived positive consequences, perceived negative consequences, and ethical considerations) as latent constructs and conducted psychometric assessments consistent with standard psychometric scale development processes to validate them (e.g., Boateng et al., 2018).

## CONCEPTUALIZING WILLINGNESS TO COEXIST WITH CARNIVORES AND ITS UNDERLYING BELIEFS

Large carnivores can play crucial ecological roles through their direct interactions with prey and indirect effects on ecosystem health (Piccoli et al., 2024; Ripple et al., 2014; Tossens et al., 2025). Their conservation, however, is complicated by human–wildlife conflict and negative perceptions. Conservation efforts have increasingly embraced human–wildlife coexistence as a framework that recognizes that successful strategies must address human behavior, beliefs, and decision‐making (Carter & Linnell, 2016; König et al., 2020; Venumière‐Lefebvre et al., 2022). This approach balances wildlife and human needs amid growing population pressures and resource demands, making it essential to understand the human actions that facilitate coexistence to develop inclusive conservation strategies that benefit both wildlife and human communities (Carter & Linnell, 2016).

Carter and Linnell (2016), for instance, describe coexistence with large carnivores in shared landscapes as a “[d]ynamic but sustainable state in which humans and large carnivores co‐adapt to living in shared landscapes where human interactions with carnivores are governed by effective institutions that ensure long‐term carnivore population persistence, social legitimacy, and tolerable levels of risk” (p. 575). Others have defined *coexistence* as an alternative to extirpation (Dickman et al., 2011) or as a reduction in the risk of human‐caused mortality to species (Oriol‐Cotterill et al., 2015). In our view, coexistence occurs when humans and wildlife share spaces with minimal conflict, including direct impacts between humans and carnivores and social conflict among people regarding carnivores (Venumière‐Lefebvre et al., 2022). Humans play a crucial role in coexistence, whether by taking proactive steps to prevent conflict, bearing some losses, sharing resources, reducing threats to carnivores, supporting species conservation, or influencing policy to advance coexistence.

Tolerance and acceptance are two other concepts related to coexistence in the conservation social science literature. Coexistence differs from tolerance and acceptance due to its active nature (Glikman et al., 2021), and its emphasis on the wildlife that are coexisted with (i.e., increasing their survival, decreasing their mortality, and helping them to thrive in the long term) (Carter & Linnell, 2016). Tolerance emphasizes passivity or inaction in the face of undesirable consequences resulting from interactions with wildlife (Bruskotter & Fulton, 2012). Likewise, acceptance usually refers to the extent to which individuals are willing to accept different levels of local wildlife populations and their associated impacts (Decker & Purdy, 1988). In contrast, coexistence requires humans to go beyond tolerance and acceptance and take actions to accommodate living with wildlife (IUCN, 2023). Consequently, we believe coexistence with carnivores is a distinct, overarching concept that transcends tolerance and acceptance.

We define *willingness to coexist* in terms of individuals’ willingness to undertake an array of actions to accommodate the existence of carnivores in the area in which they live. Willingness to act is a close proxy for actual behavior (Ajzen, 1991; Pomery et al., 2009). Human–wildlife coexistence involves behaviors that span personal and collective domains (Carter & Linnell, 2016). This distinction parallels frameworks in environmental psychology that differentiate private‐ and public‐sphere behaviors (Stern, 2000), but it is also reflected in other fields. Political theory emphasizes the distinction between individual and civic duties through the concept of environmental citizenship (Dobson, 2007), and research on political consumerism highlights how personal consumption choices can also serve as collective political acts (Micheletti, 2003). Building on this literature, we conceptualized coexistence behaviors as comprising individual actions (e.g., individual adaptations to reduce conflict) and collective actions (e.g., policy support and civic engagement). We recognized that both domains allow for a more comprehensive assessment of coexistence and its underlying drivers.

We conceptualized beliefs about coexistence as cognitive and moral antecedents that shape individuals’ willingness to coexist with carnivores. We treated beliefs and willingness as distinct latent constructs and validated them on separate scales. Our theoretical framework for understanding cognitive psychological drivers of coexistence with large carnivores rests on two foundations: the relevant conservation literature and sociopsychological theories. The first draws from risk perception theory (Slovic, 1987), which posits that a risk–benefit assessment process determines acceptance of potential hazards (Alhakami & Slovic, 1994; Siegrist, 1999). Risk acceptance is determined through a cognitive assessment process in which individuals weigh perceived benefits against risks, increasing acceptance when benefits are salient and personally relevant (Fischhoff et al., 1978). Studies demonstrate that tolerance for large carnivores increases when perceived benefits outweigh perceived risks to human lives and livelihoods (Bruskotter & Wilson, 2014; Carter et al., 2012). This relationship has been documented for existing and reintroduced carnivore populations. Communicating species benefits can enhance public acceptance (Slagle et al., 2013; Toth & Rubino, 2024), whereas perceived risks from carnivores decrease support (Adams Knopff et al., 2016; Ghasemi et al., 2021; Mcgovern & Kretser, 2015).

The second theoretical foundation draws from research in moral psychology and decision science that shows ethical considerations can influence attitudes and behavioral intentions independent of utilitarian cost–benefit assessments (Bartels, 2008; Bennis et al., 2010). Moral evaluations can precede and influence perceptions of costs and benefits, even when individuals lack direct information about consequences (Liu & Ditto, 2013; Sparks & Shepherd, 2002). This moral dimension has emerged as significant in wildlife conservation, where moral considerations (e.g., species’ intrinsic value and ethical obligations) can influence conservation behavior and attitudes (Batavia et al., 2018; Bruskotter et al., 2019; Ghasemi & Kyle, 2021; Lute et al., 2016; Pooley et al., 2021; Vucetich et al., 2015). Moral framing of carnivore conservation, particularly emphasizing ethical responsibilities to restore extirpated species, can enhance public support for carnivore presence and recovery programs (Hermann & Menzel, 2013). Moreover, attributing intrinsic value to animals can fundamentally reshape human–wildlife relationships and increase tolerance for their presence (Lute et al., 2016). Table 1 contains the definitions of the constructs we used.

**TABLE 1:** Definitions of constructs conceptualized for willingness to coexist with large carnivores and its underlying beliefs.

Construct	Definition
Willingness to coexist	Individuals’ willingness to undertake an array of actions to accommodate the existence of carnivores in the area in which they live
Individual domain	Personal‐ or private‐sphere dimension of willingness, referring to individual actions or adaptations (e.g., modifying behavior, protecting property) that enable coexistence with carnivores
Collective domain	Public‐sphere dimension of willingness, referring to civic or community‐level actions (e.g., supporting policies, management strategies, or collective initiatives) that facilitate coexistence
Beliefs about coexistence	Underlying cognitive and moral appraisals that shape willingness to coexist, composed of evaluations of consequences and ethical considerations of living with carnivores
Perceived positive consequences	Beliefs about the benefits of carnivore presence, such as ecological roles, cultural values, or economic opportunities (e.g., tourism)
Perceived negative consequences	Beliefs about the risks or costs of carnivore presence, such as threats to safety, livelihoods, property, or pets
Ethical considerations	Moral judgments regarding the rightness or wrongness of allowing carnivores to persist, including beliefs about their intrinsic value and humans’ responsibilities to them

## PSYCHOMETRIC SCALE DEVELOPMENT

Psychometrics, a branch of psychology, focuses on measuring mental abilities and processes, including personality traits and emotional states (Nunnally & Bernstein, 1994). Two key criteria in evaluating psychological instruments, such as questionnaires, are validity and reliability. Validity refers to the extent to which an instrument measures what it is intended to measure, ensuring accuracy, whereas reliability indicates the consistency of measurement over time and across respondents (Price, 2017). Ensuring both validity and reliability is essential for producing trustworthy and generalizable findings, thereby advancing understanding of human attitudes and behaviors in biodiversity conservation (Kyle et al., 2020; Nunnally & Bernstein, 1994; Whitehouse‐Tedd et al., 2021). To ensure validity and reliability, scale development follows a systematic process of construct conceptualization, item generation, and empirical validation (Hinkin, 1995; Price, 2017). Initial steps involve developing a theoretical framework and creating a comprehensive item pool. Subsequent psychometric evaluation encompasses reliability assessment through internal consistency and test–retest analyses (Boateng et al., 2018; Price, 2017).

**FIGURE 1:**
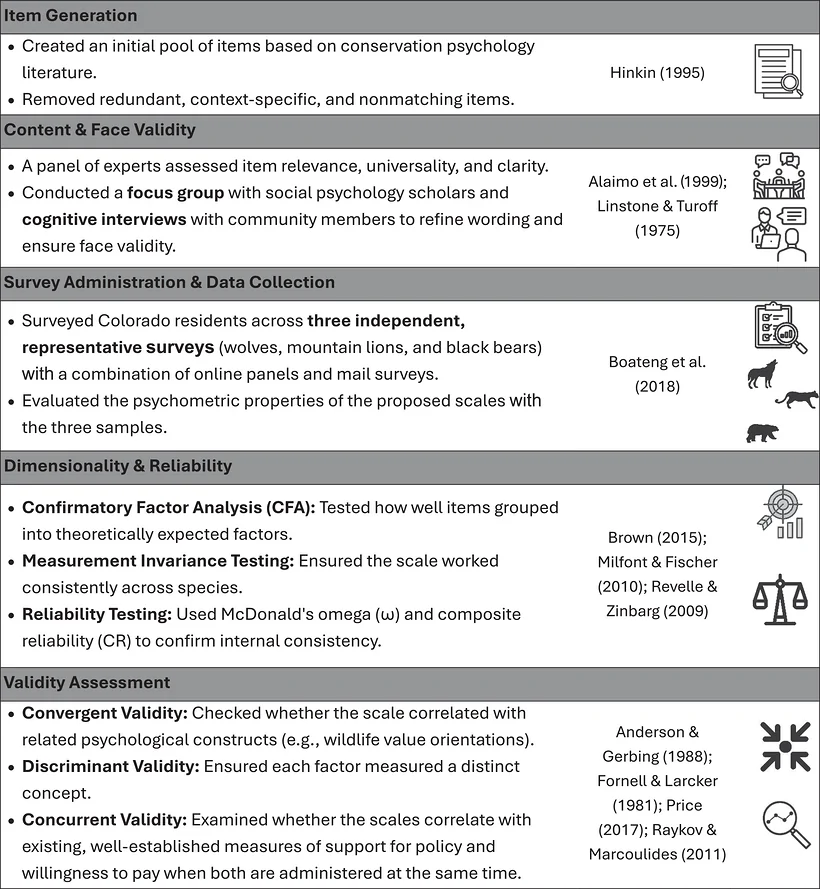
Overview of the psychometric scale development process used to validate the willingness to coexist and beliefs about coexistence scales.

We followed scale development recommendations through an iterative process of expert reviews for theoretical refinement and item generation, followed by cognitive interviews and rigorous statistical analyses with data from three case studies to ensure measurement validity and reliability. Our scale development and validation followed recommendations from Boateng et al. (2018), Kyle et al. (2020), and Price (2017). An overview of the process is shown in Figure 1. Data were analyzed using SPSS 29 and Mplus 8.11, and model fit was assessed according to Hair et al. (2014). We employed full‐information maximum likelihood estimation for all the measurement invariance and confirmatory factor analyses (CFAs). No violation of data normality was observed.

### Item generation, content validity, and face validity

First, we established an item pool with previously validated measures (Hinkin, 1995). We removed items that were incongruent with construct definitions, redundant, or context specific. We conducted a literature search in Google Scholar and Web of Science for terms related to our proposed constructs and generated an initial pool of items extracted from 35 studies (Appendix S1). After removing redundant, context‐specific, and nonmatching items, 48 remained (Appendix S2).

Fourteen academic and practitioner experts in human–carnivore interactions, representing natural and social sciences (including the authors of this paper), assessed content and face validity of the scales through an iterative Delphi process (Linstone & Turoff, 1975) to ensure relevance, universality, and clarity. The experts’ perspectives were informed by extensive professional experience in human–wildlife coexistence across multiple regions and species in collaboration with communities, conservation organizations, academia, and government agencies. Using a 5‐point scale (1 = *terrible* to 5 = *excellent*), the experts evaluated the items based on three criteria: relevance to the construct (with definitions provided), universality (applicability across species, cultures, regions, and stakeholder groups), and clarity (face validity). Experts also suggested additional universal items. Final item selection was based on the ratings for relevance, universality, and clarity. Further details on the expert review are in Appendix S2. Based on expert feedback and ratings, we refined the item wording and selected eight items for WCoEx individual, six for WCoEx collective, and six for each of the perceived negative consequences, perceived positive consequences, and ethical considerations, resulting in 32 final items.

Next, we conducted cognitive interviews with citizens (*n* = 5) and a focus group with social psychology scholars and graduate students (*n* = 8) to ensure face validity, refine item wording, and finalize item structure (Alaimo et al., 1999). These items were further refined based on feedback from the cognitive interviews and focus group discussions. The final list of items and their wording is in Table 3. The WCoEx items were rated on a 5‐point scale, ranging from *not at all willing* (0) to *extremely willing* (4). Beliefs about coexistence items were measured on a 5‐point Likert‐type scale, ranging from *strongly disagree* (−2) to *strongly agree* (+2).

### Study samples

We validated our proposed scales with data from three samples in 2022. People surveyed on gray wolves (*Canis lupus*) (*n* = 434) were identified from an online panel of Colorado residents using the probability‐based KnowledgePanel (Hoag et al., 2023). Participants surveyed on mountain lions (*Puma concolor*) (*n* = 442) and black bears (*Ursus americanus*) (*n* = 433) were identified through surveys on species management that used stratified random sampling with mailed invitations to home addresses (Ghasemi et al., 2024). The mountain lion and black bear surveys were mutually exclusive. All respondents were Colorado residents aged 18 or older. Data were collected using mail‐back and online surveys for the mountain lion and black bear samples. For the survey on wolves, an online‐only data collection method was used. All online survey instruments were hosted on the Qualtrics online survey platform. The Colorado State University Institutional Review Board exempted the study from a full review process and approved the survey instruments and data collection protocols (scale development process, protocol 2921; survey on wolves, protocol 2986; survey on mountain lions and black bears, protocol #3180).

The ecological status and management of wolves, black bears, and mountain lions in Colorado illustrate diverse conservation scenarios. At the time we collected data (2022), wolves were listed as endangered at the federal and state levels in Colorado, but they had not yet been reintroduced in the state, where they had been irradicated in the 1940s. In late 2023, following a narrowly approved citizen‐initiated ballot initiative in 2020, wolves were reintroduced through efforts led by Colorado Parks and Wildlife and the U.S. Fish and Wildlife Service (CPW, 2023). Colorado is estimated to host 17,000–20,000 black bears (CPW, 2015) and 3800–4400 mountain lions (excluding kittens) (CPW, 2024). These species frequently interact with humans in urban and rural areas. Their core habitat is in the Rocky Mountains. Neither black bears nor mountain lions were listed as endangered species when we collected data.

### Dimensionality

Because our primary objective was scale development and validation, beliefs about coexistence and willingness to coexist were analyzed as separate measurement models rather than within a structural model specifying directional effects. We tested the predefined factor structures based on theoretical foundations with CFA (Brown, 2015). To test the dimensionality of our proposed measures, we evaluated three different hypothesized measurement models with structural equation modeling (Appendix S3): a single‐factor model with all the items related to one construct (i.e., willingness to coexist or beliefs about coexistence); a multifactor model with items related to each of the hypothesized dimensions of the constructs, with the dimensions correlated to each other; and a second‐order factor model based on the assumption that any correlation between the primary factors (or dimensions) is due to a more abstract construct. We compared the model goodness‐of‐fit statistics to select the dimensionality that best described the theoretical structure of our constructs.

For WCoEx, we analyzed three different measurement models (Appendix S3). The first model had one factor with all 14 individual and collective WCoEx items loaded onto it. The second model had two correlated factors corresponding to the individual and collective dimensions; the related items were loaded onto each. Finally, the third model assumed a second‐order abstract factor that best accounts for the variation among the two dimensions of individual and collective WCoEx. Model fit indices (Table 2) indicated that the two‐factor model provided the best representation of the data, with superior χ^2^, root mean square error of approximation (RMSEA), comparative fit index (CFI), and Tucker–Lewis index (TLI) values across all three species.

**TABLE 2:** Goodness of fit of measurement models used to test dimensionality of willingness to coexist and beliefs about coexistence constructs^a^.

Model		χ^2^ (df)	CFI	TLI	RMSEA [90% CI]
Willingness to coexist					
One factor	Wolf	316.52 (72)	0.96	0.94	0.089 [0.079‐0.099]
	Mountain lion	377.12 (72)	0.95	0.93	0.098 [0.088–0.108]
	Black bear	414.96 (72)	0.94	0.91	0.105 [0.095–0.115]
Two factors	Wolf	273.15 (71)	0.97	0.95	0.081 [0.071–0.091]
	Mountain lion	254.03 (71)	0.97	0.96	0.076 [0.066–0.087]
	Black bear	288.80 (71)	0.96	0.94	0.084 [0.074–0.095]
Second order	Wolf	273.15 (71)	0.97	0.95	0.081 [0.071–0.091]
	Mountain lion	254.03 (71)	0.97	0.96	0.076 [0.066–0.087]
	Black bear	288.80 (71)	0.96	0.94	0.084 [0.074–0.095]
Beliefs about coexistence					
One factor	Wolf	457.80 (113)	0.95	0.92	0.084 [0.076−0.092]
	Mountain lion	629.01 (113)	0.92	0.88	0.102 [0.094−0.110]
	Black bear	613.47 (113)	0.90	0.85	0.101 [0.093−0.109]
Three factors	Wolf	182.98 (110)	0.99	0.98	0.039 [0.029−0.049]
	Mountain lion	244.05 (110)	0.98	0.97	0.053 [0.044−0.061]
	Black bear	199.82 (110)	0.98	0.97	0.043 [0.034−0.053]
Second order	Wolf	197.73 (111)	0.99	0.98	0.042 [0.033−0.052]
	Mountain lion	245.92 (111)	0.98	0.97	0.052 [0.044−0.061]
	Black bear	206.03 (111)	0.981	0.971	0.045 [0.035−0.054]

^a^ Wolf survey (*n* = 434), mountain lion survey (*n* = 442), and black bear survey (*n* = 433).

Abbreviations: CFI, comparative fit index; CI, confidence interval; RMSEA, root mean square error of approximation; TLI, Tucker–Lewis index.

Similarly, for beliefs about coexistence, we estimated three measurement models: all items loading on the same factor, three correlated factors (i.e., perceived positive consequences, perceived negative consequences, and ethical considerations), and the second‐order factor model (Appendix S3). The three correlated factor model showed the best fit to the data across the three samples (Table 2), indicating that the attitudinal scales are better represented as three correlated factors rather than a second‐order factor model. The variation among the indicators was best captured by three correlated first‐order constructs rather than a superordinate, second‐order latent construct (Table 2). Item factor loadings of the selected models for WCoEx and beliefs about coexistence are in Table 3.

**TABLE 3:** Item factor loadings and psychometric scale reliability coefficients of the final scale items used in willingness to coexist and beliefs about coexistence scales.

Construct and item^a^	λ^b^			CR^c^			ω^d^		
	Wolf	Mt. lion	Black bear	Wolf	Mt. lion	Black bear	Wolf	Mt. lion	Black bear
Willingness to coexist (WCoEx)										
Individual (Ind)				0.90	0.90	0.89	0.90	0.92	0.91
WCoEx‐Ind1—Change my behavior to reduce potential conflicts between people and wolves, mountain lions, or black bears.	0.84	0.80	0.75						
WCoEx‐Ind2—Accept some negative impact from wolves/mountain lions/black bears on my livelihood or source of income.	0.74	0.68	0.65						
WCoEx‐Ind3—Learn about ways to reduce potential conflicts between people and wolves/mountain lions/black bears.	0.79	0.77	0.79						
WCoEx‐Ind4—Oppose killing a wolf/mountain lion/black bear unless my personal or family's safety is at risk.	0.67	0.67	0.60						
WCoEx‐Ind5—Oppose killing a wolf/mountain lion/black bear unless my animals (pets, livestock, etc.) are at risk.	0.59	0.68	0.57						
WCoEx‐Ind6—Oppose killing a wolf/mountain lion/black bear unless my property (home, vehicle, etc.) is at risk.	0.42	0.63	0.63						
WCoEx‐Ind7—Find ways to protect my livestock or property from wolves/mountain lions/black bears without harming the wolf/mountain lion/black bear.	0.81	0.79	0.84						
WCoEx‐Ind8—Find ways to protect myself, my family, or my pets from wolves/mountain lions/black bears without harming the wolf/mountain lion/black bear.	0.80	0.82	0.82						
Collective (Col)				0.96	0.96	0.96	0.96	0.96	0.95
WCoEx‐Col1—Support policies and management actions to conserve wolves/mountain lions/black bears.	0.93	0.89	0.89						
WCoEx‐Col2—Support the efforts of wildlife management authorities working to help people share the landscape with wolves/mountain lions/black bears.	0.93	0.94	0.94						
WCoEx‐Col3—Encourage wildlife management authorities to promote ways to share the landscape with wolves/mountain lions/black bears.	0.92	0.94	0.94						
WCoEx‐Col4—Encourage my friends and family to promote ways to share the landscape with wolves/mountain lions/black bears.	0.91	0.93	0.93						
WCoEx‐Col5—Work with my community to promote ways to share the landscape with wolves/mountain lions/black bears.	0.90	0.88	0.88						
WCoEx‐Col6—Contribute time or money to programs that help people share the landscape with wolves/mountain lions/black bears.	0.82	0.73	0.73						
Beliefs about coexistence										
Perceived positive consequences (PPO)				0.93	0.93	0.93	0.95	0.94	0.92
Having wolves/mountain lions/black bears in the area where I live would…									
PPO1—benefit me.	0.79	0.79	0.79						
PPO2—be good for the overall health of the environment.	0.89	0.89	0.89						
PPO3—benefit my community.	0.87	0.85	0.85						
PPO4—improve my quality of life.	0.80	0.78	0.78						
PPO5—benefit other animals and plants.	0.89	0.83	0.83						
PPO6—be an important part of my culture.	0.77	0.80	0.80						
Perceived negative consequences (PNO)				0.89	0.91	0.91	0.91	0.92	0.91
Having wolves/mountain lions/black bears in the area where I live would…									
PNO1—threaten my personal or family's safety.	0.79	0.83	0.83						
PNO2—threaten the safety of my animals (pets, livestock, etc.).	0.75	0.72	0.72						
PNO3—threaten my property, livelihood, or income.	0.76	0.80	0.80						
PNO4—limit my ability to use my land the way I want.	0.75	0.80	0.80						
PNO5—threaten livestock or agricultural production in my community.	0.65	0.76	0.76						
PNO6—threaten local outdoor recreation opportunities (recreational hunting, bird watching, hiking, etc.).	0.80	0.84	0.84						
Ethical considerations (ETH)				0.89	0.89	0.89	0.92	0.90	0.87
ETH1—It would be important to maintain wolves/mountain lions/black bears in my area for future human generations.	0.94	0.88	0.88						
ETH2—Whether or not I get to see wolves/mountain lions/black bears, it would be important to know that they exist in my area.	0.75	0.71	0.71						
ETH3—Wolves/mountain lions/black bears deserve to be conserved for their own value.	0.78	0.77	0.77						
ETH4—Individual wolves/mountain lions/black bears should not be harmed by humans without cause.	0.62	0.62	0.62						
ETH5—Wolves/mountain lions/black bears have value, above and beyond their use to humans.	0.74	0.75	0.75						
ETH6—Wolves/mountain lions/black bears should exist whether they serve human needs or not.	0.74	0.75	0.75						

^a^ The WCoEx items are rated on a 5‐point scale ranging from 0 = *not at all willing* to 4 = *extremely willing*. Beliefs about coexistence items are measured using a 5‐point Likert‐type scale ranging from −2 = *strongly disagree* to +2 = *strongly agree*.

^b^ Standardized factor loading.

^c^ Composite reliability.

^d^ McDonald's omega.

### Measurement invariance

We conducted measurement invariance testing on the three surveys on carnivores (wolves, black bears, and mountain lions) to assess whether the measurement model's psychometric properties can be generalized across different species (Byrne et al., 1989; Milfont & Fischer, 2010). We estimated the unconstrained measurement models simultaneously across the three samples. The goodness‐of‐fit indices indicated good model fit to the data and supported configural invariance. The fit statistics of the unconstrained models served as the baseline for comparing the fit of the following constrained models, where, in each step, we increasingly imposed model constraints by setting parameters equal across groups.

We constrained the measurement intercepts to be equal across samples to test scalar invariance. This constraint did not result in a significant decline in model fit compared with the baseline models (ΔCFI < 0.01 and all RMSEA confidence intervals overlapped) (Cheung & Rensvold, 2002). We concluded the invariance of the WCoEx and beliefs about coexistence models across the three samples. Results indicated configural, factorial, and scalar invariance of our measurement model across the wolf, mountain lion, and black bear samples (Appendix S4). These results confirmed that the scale can be used across the tested species without bias, enabling comparisons across different conservation contexts.

### Reliability

Scale reliability was assessed using McDonald's omega (ω) and composite reliability, both superior to Cronbach's alpha (Revelle & Zinbarg, 2009). Across scales and samples, both composite reliability and McDonald's omega coefficients were above the cutoff criteria for good scale reliability (>0.8) (Lance et al., 2006) (Table 3). These results indicated internal consistency of the proposed scales across the three carnivore species samples.

### Convergent validity

Convergent validity is evidence of construct validity, which reflects alignment with theoretically related latent constructs (Price, 2017). It can be assessed by verifying that each scale accounted for over 50% of the variance in its indicators (average variance explained [AVE] >0.5 [Hair et al., 2014]) and through correlations with theoretically related constructs measured concurrently in the same surveys (Raykov & Marcoulides, 2011). We computed AVEs for the proposed scales, and all the proposed scales had AVE values >0.5, which provided further evidence of convergent validity (Table 5) (Hair et al., 2014). We examined the scales’ correlations with other constructs to which they were theoretically related (Table 4). Respondents were presented with items examining their wildlife value orientations (across three samples [Manfredo et al., 2009]) and trust in the state wildlife agency (Needham & Vaske, 2008) (wolf sample only) (Appendix S5). The proposed scales were moderately to highly correlated with wildlife value orientations (across samples) and with trust in the agency (wolf sample only).

**TABLE 4:** Bivariate correlations (Pearson's) between the proposed psychometric scales and theoretically related external constructs to evaluate convergent and concurrent validity of willingness to coexist and beliefs about coexistence scales.

	Wolf sample (*n* = 434)					Mountain lion sample (*n* = 442)				Black bear sample (*n* = 433)			
	WVO mutualism	WVO domination	Trust in agency	Willingness to pay	Reintroduction approval	WVO mutualism	WVO domination	Future population	Preventive measures	WVO mutualism	WVO domination	Future population	Preventive measures
Willingness to coexist													
Individual	0.54	−0.49	0.54	0.37	0.57	0.62	−0.53	0.44	0.61	0.60	−049	0.44	0.60
Collective	0.61	−0.52	0.65	0.44	0.71	0.65	−0.55	0.49	0.60	0.60	−0.42	0.43	0.64
Beliefs about coexistence													
Perceived negative consequences	−0.43	0.48	−0.45	−0.36	−0.57	−0.38	0.39	−0.42	−0.35	−0.25	0.31	−0.31	−0.33
Perceived positive consequences	−0.50	0.54	0.54	0.41	0.72	0.56	−0.44	0.51	0.43	0.45	−0.34	0.48	0.48
Ethical considerations	0.62	0.60	0.60	0.42	0.70	0.62	−0.53	0.49	0.51	0.58	−0.46	0.41	0.55

*Note*: Pearson's correlations are statistically significant at *p* < 0.01. Composite scores were calculated for multiple‐item constructs by averaging the indicators. All multiple‐item constructs were reliable (ω ≥ 0.73) (Appendix S5).

Abbreviation: WVO, wildlife value orientations.

### Concurrent validity

Concurrent validity is a form of criterion‐related validity, reflecting associations with applied or behavioral indicators measured at the same time (Price, 2017). We evaluated the concurrent validity of our proposed scales by examining their correlations with constructs measured in the same surveys, indicating some real‐world outcomes of coexistence. For the survey on wolves, we included willingness to pay for wolf reintroduction and approval of wolf reintroduction as indicators of coexistence. Willingness to pay for wolf reintroduction was estimated from a choice experiment conducted as part of the same survey (Hoag et al., 2023). Wolf reintroduction approval was gauged using a single item that asked respondents about their approval of wolf reintroduction in Colorado following the ballot initiative that occurred before the survey.

For the surveys on mountain lions and black bears, we included respondents’ willingness to undertake conflict‐preventive measures for carnivores and their wildlife acceptance capacity (i.e., future carnivore population preference; Decker & Purdy, 1988), measured in the same survey, as indicators of coexistence. Preventive measures included actions that residents could adopt to reduce the risk of conflict with carnivores. For wildlife acceptance capacity, we asked respondents whether they would like mountain lion or black bear populations in Colorado to decrease or increase in the next 5 years. Further details about these variables are in Appendix S5. All our proposed scales displayed medium to strong correlations (>0.3) (Hair et al., 2014) in the anticipated direction (Table 4). These results provide evidence for the concurrent validity of these scales.

### Discriminant validity

Discriminant validity (i.e., the extent to which a measure is statistically distinct from other constructs, ensuring it does not simply reflect a different variable [Price, 2017]) can be evaluated using the Fornell and Larcker (1981) criterion: AVE must be greater than squared correlations between constructs. Another criterion requires constraining correlations between latent factors to 1.0 and using chi‐square difference testing to see whether this constraint leads to significant model fit deterioration (Anderson & Gerbing, 1988; Byrne, 1998). We checked whether the AVE values for each pair of constructs in the measurement model were greater than the squared correlation between them (Fornell & Larcker, 1981). According to this criterion, all the proposed scales showed discriminant validity except for perceived positive consequences and ethical considerations in the survey on wolves, ethical considerations in the survey on mountain lions, and WCoEx individual across all three surveys (Table 5).

**TABLE 5:** Evidence for convergent and discriminant validity of psychometric scales to measure willingness to coexist and beliefs about coexistence with large carnivores.

		Mean variance	1	2	3
Willingness to coexist					
Individual	Wolf	0.53	–	Δχ^2^ = 27.44	
	Mountain lion	0.54		Δχ^2^ = 55.81	
	Black bear	0.52		Δχ^2^ = 56.58	
Collective	Wolf	0.81	*r* = 0.95	–	
	Mountain lion	0.80	*r* = 0.88		
	Black bear	0.77	*r* = 0.85		
Beliefs about coexistence					
Perceived positive consequences	Wolf	0.70	–	Δχ^2^ = 3250.11	Δχ^2^ = 46.41
	Mountain lion	0.68		Δχ^2^ = 2781.14	Δχ^2^ = 61.27
	Black bear	0.62		Δχ^2^ = 2111.62	Δχ^2^ = 75.83
Perceived negative consequences	Wolf	0.57	*r* = −0.74	–	Δχ^2^ = 2514.71
	Mountain lion	0.63	*r* = −0.69		Δχ^2^ = 2200.57
	Black bear	0.60	*r* = −0.66		Δχ^2^ = 1126.93
Ethical considerations	Wolf	0.59	*r* = 0.87	*r* = −0.69	–
	Mountain lion	0.56	*r* = 0.81	*r* = −0.65	
	Black bear	0.50	*r* = 0.70	*r* = −0.50	

*Wolf survey (*n* = 434), mountain lion survey (*n* = 442), and black bear survey (*n* = 433). Values below the diagonal represent correlations (*r*) obtained from the measurement model estimations. Values above the diagonal represent chi‐square differences (Δdf = 1) for the latent factor correlation constraint test. All tests were statistically significant at *p* < 0.001.

In the second method, we constrained the correlation between each pair of latent factors to be equal to 1 (resulting in the drop of one degree of freedom) and examined the effect on model fit with the chi‐square difference test (Anderson & Gerbing, 1988; Byrne, 1998). As shown in Table 5, in all cases, the constrained model demonstrated a statistically significant decline in model fit (all at *p* < 0.001), indicating the discriminant validity of our scales.

## DISCUSSION

Recognizing that successful carnivore conservation hinges on human acceptance and behavior, we addressed a critical methodological gap in measuring human willingness to coexist with these species. By developing and validating two distinct psychological scales for WCoEx and its underlying coexistence beliefs, we provide standardized, psychometrically robust instruments that can offer conservation researchers and practitioners a reliable means of monitoring and evaluating human–carnivore coexistence. This responds to growing calls for stronger social science methods in conservation and supports the emerging coexistence paradigm that seeks to balance human and wildlife needs (Carlson et al., 2023; Carter & Linnell, 2016; Moon & Blackman, 2014; Teel et al., 2018). Our findings demonstrate that the WCoEx and coexistence belief scales exhibit strong reliability and validity across multiple carnivore species, highlighting their potential utility for both research and applied conservation and underscoring the need for continued testing and adaptation.

### Theoretical implications

Our findings suggest that willingness to coexist comprises two distinct but related dimensions: individual and collective behaviors. This distinction enhances understanding of human–wildlife coexistence by revealing how individuals accommodate carnivores differently in their personal versus social contexts. Individual behaviors involve personal modifications to reduce conflict (e.g., nonlethal protection of livestock), whereas collective behaviors involve supporting broader social and political efforts (e.g., contributing to conservation or advocating for nonlethal policies). The strong correlation between these dimensions suggests a shared underlying psychological construct while remaining empirically distinct, aligning with environmental psychology research on private‐ and public‐sphere behaviors (Dobson, 2007; Micheletti, 2003; Stern, 2000). Recognizing this separation is a step in building upon existing coexistence frameworks (Carter & Linnell, 2016) because it suggests that individual adaptation and social institutions operate through separate psychological pathways. Furthermore, this correlation suggests a spillover effect from private to public action (Thøgersen & Ölander, 2003). For instance, an individual who adopts nonlethal methods at home may become more likely to support collective carnivore‐friendly policies. Therefore, successful coexistence interventions should be designed to foster initial individual behavioral change, thereby encouraging subsequent engagement in broader collective support.

Our distinction between individual and collective willingness provides a foundation for future research to examine contextual barriers and enabling conditions for coexistence behaviors. For example, individual willingness may be constrained by limited resources or knowledge, whereas collective willingness may depend on governance structure, political will, or institutional support (Stern, 2000). Identifying these contextual factors could help refine the scale and inform interventions that target both individual adaptations and broader governance processes.

We analyzed beliefs and willingness in separate measurement models to meet psychometric best practices for scale development, rather than as indicators within a single higher‐order construct. This analytic choice reflects our goal of establishing the reliability and validity of each scale independently before testing directional or mediational relationships in future research. Moreover, the validation of the coexistence belief scale and its three dimensions (i.e., perceived positive consequences, perceived negative consequences, and ethical considerations) supports our framework, integrating risk perception and moral psychology. The model fit and discriminant validity indicated that beliefs about carnivore coexistence involved distinct cognitive processes for evaluating benefits, risks, and moral obligations. This reinforces prior findings that utilitarian and moral considerations shape conservation attitudes (Batavia et al., 2018; Lute et al., 2016) and demonstrates that they are separate constructs.

Identifying ethical considerations as a distinct factor provides a more holistic view of human judgment and decision‐making than risk–benefit models of human–wildlife interactions imply (Pooley et al., 2021). This supports past research (Lute et al., 2016; Vucetich et al., 2015), suggesting moral obligations toward wildlife function independently of utilitarian reasoning. Our results suggest that ethical frameworks may provide an alternative means of promoting coexistence, particularly when benefit‐based arguments are insufficient, and align with evidence that moral framing can enhance conservation support, even among those who perceive high risks (Hermann & Menzel, 2013).

### Methodological contributions

Following best practices in psychometric research, our scale development process produced instruments with strong reliability and various forms of validity. The high composite reliability and McDonald's omega coefficient values across all scales and species samples indicated internal consistency. At the same time, significant correlations with theoretically related constructs supported concurrent and convergent validity. These results suggest that our scales provide precise, stable measurements of their intended constructs.

Measurement invariance across surveys on wolves, mountain lions, and black bears demonstrated that willingness to coexist and its underlying beliefs were measured equivalently across species. This is particularly important given these carnivores’ differing ecological roles, cultural meanings, and management histories, which could otherwise confound comparisons. Demonstrating configural, metric, and scalar invariance is an essential but rarely applied step in human dimensions of human–wildlife coexistence research (Milfont & Fischer, 2010). Our results support meaningful cross‐species comparisons, including comparisons of mean levels of willingness to coexist. This strengthens confidence in the scale's theoretical generalizability and utility for comparative research and monitoring and represents an initial step toward external validity that requires further testing across additional taxa and diverse cultural and geographic contexts.

The somewhat weaker discriminant validity between WCoEx subscales warrants further exploration. Although the chi‐square difference tests supported discriminant validity, the AVE criterion suggested some overlap between WCoEx individual and WCoEx collective. It is common for different tests to yield mixed results because each method has varying sensitivities. Although some metrics fell short, our use of multiple tests strengthened confidence in the scales. The overall findings are promising; however, further validation is needed. The strong connection between the two dimensions of willingness to coexist might reflect the inherently interconnected nature of individual and collective behaviors in conservation contexts, where individual actions often have community‐level implications.

The mixed evidence for discriminant validity of our constructs may also reflect common method variance in survey research, which can lead to inflated correlations between constructs (Baumgartner et al., 2021). Acquiescence bias (i.e., the tendency to agree with items regardless of content) could partially explain the high correlations between constructs (Podsakoff et al., 2003). Future studies could address this by incorporating marker variables, which are theoretically unrelated to the focal constructs but measured in the same way (Richardson et al., 2009).

### Practical implications

The validated WCoEx scale provides conservation practitioners with a standardized tool for assessing and monitoring social capacity for human–carnivore coexistence. Recent research has highlighted the importance of rigorous methods in understanding sociopsychological factors that contribute to conservation success (Fox et al., 2006; Moon et al., 2019; Whitehouse‐Tedd et al., 2021), and our scale provides a validated instrument for this purpose. For example, wildlife managers working on carnivore reintroduction or restoration programs can use the scales to assess communities’ willingness to coexist and track changes over time and adapt their management accordingly (Lecuyer et al., 2022; Lute et al., 2020; Niemiec et al., 2021).

The distinction between individual and collective dimensions can inform the design of interventions across various contexts. For ranchers and other local communities directly interacting with carnivores, programs may focus on individual behaviors, such as adopting conflict prevention measures, drawing on successful examples documented by Carter et al. (2012) and Slagle et al. (2013). In urban areas, efforts may emphasize collective behaviors that support procarnivore conservation policies and governance (Carter & Linnell, 2023).

The validation of separate coexistence belief scales offers practical guidance for tailoring communication strategies (Kidd et al., 2019). For instance, when addressing perceived negative consequences, managers might adopt the approach outlined by Slagle et al. (2013), which combines technical risk information with benefit messaging. The distinct ethical considerations scale suggests that incorporating moral framing, such as emphasizing the intrinsic value of species and the rights of future generations to experience wildlife, is valuable.

Practitioners can use these scales to identify specific barriers to coexistence in their communities. If ethical considerations are conducive but perceived risks dominate behavior, resources might be best directed toward practical risk mitigation measures. Conversely, low ethical consideration scores may indicate a need for educational programs that emphasize the ecological, intrinsic, and cultural values of wildlife (e.g., Toth & Rubino, 2024).

The scales’ cross‐species validity enables consistent monitoring across diverse conservation contexts. Managers working with multiple carnivore species can use the same instrument to assess community beliefs and willingness to coexist, facilitating comparison of intervention effectiveness across species and regions. The standardized scales we developed address the need identified for comparable psychological measures in conservation psychology (Kyle et al., 2020; Whitehouse‐Tedd et al., 2021).

### Limitations and future research

This study has several limitations that suggest directions for future research. Our scales were designed to be broadly applicable, and our approach was built on broader practices and research, including items that have been tested previously in different contexts. Nonetheless, we validated our proposed scales based on representative samples of Colorado residents; thus, further testing across diverse geographic, sociopolitical, and cultural contexts is needed to confirm their generalizability (Teel et al., 2007).

Our framework represents an a priori conceptualization of willingness to coexist and its underlying beliefs, informed by theory and literature, and emphasizes cognitive and moral dimensions grounded in Western scientific traditions. As such, it may not fully capture the values, responsibilities, and relational worldviews that shape human–wildlife interactions in other contexts, including those of Indigenous peoples and communities guided by epistemologies centered on reciprocity, kinship, or custodianship. Our intention is not to claim universality but to provide a theoretically grounded approach that can be tested, adapted, or complemented by alternative frameworks in future research.

Future work should explore culturally specific adaptations or entirely new conceptualizations to ensure inclusive and context‐sensitive measures of coexistence. For the coexistence belief scale, although our focus was on cognitive and moral predictors of willingness to coexist, other important social and psychological factors warrant attention. For instance, trust in institutions, such as government agencies or wildlife authorities, and affective dimensions, such as emotions toward wildlife, influence conservation attitudes and behaviors (e.g., Ghasemi et al., 2021). Incorporating these variables would not only broaden the conceptual foundation but also enhance the explanatory power and practical utility of the framework for informing conservation interventions.

Hunting and trapping were not included in the WCoEx items because these practices are more specific to Western wildlife management contexts, and we aimed to develop scales that are as universal as possible. Hunting and trapping can shape how coexistence is understood in some socioecological and cultural settings, which may help explain the lower loadings of items implying categorical opposition to killing in our Colorado sample. Future research should examine whether incorporating lethal management practices improves the relevance and validity of coexistence measures in contexts where they are integral.

Although validated across three large carnivore species, the WCoEx scale's applicability to other taxa, particularly those posing different risks, perceived as pests, or holding distinct cultural meanings, remains to be tested. Our validation relied on self‐reported intentions and external variables measured concurrently with the scale, providing stronger evidence of concurrent rather than predictive validity. Future research should assess the scale's ability to predict subsequent behaviors or policy‐relevant actions to more fully establish criterion validity and continue refining the scale structure where construct overlap remains. Finally, although willingness to coexist is a theoretically grounded proxy for behavior, actual actions are shaped by contextual, social, and capability factors. Consistent with the theory of planned behavior (Ajzen, 1991) and behavioral willingness research (Pomery et al., 2009), willingness is critical but insufficient, which highlights the need to pair these measures with observed behaviors and contextual data.

## CONCLUSION

This study represents an important first step in developing a standardized, psychometrically robust measure of willingness to coexist with carnivores. The WCoEx and coexistence belief scales demonstrated strong reliability and validity across three large carnivore species in Colorado, offering a solid foundation for both research and applied use. Although further validation across other taxa, geographies, and cultural contexts is needed, our findings highlight the scale's potential as a valuable tool for conservation researchers and practitioners to monitor and evaluate human–carnivore coexistence.

## Supporting information



Supporting Information
