# Supplementary material for: Psychometric framework for coexistence with large carnivores
Source: Conserv Biol. 2026 May 14;40(5):e70326. doi: 10.1111/cobi.70326 (PMC13613547; doi:10.1111/cobi.70326)
Supplement: Supplementary file 1 [file COBI-40-e70326-s001.docx]

# Toward Coexistence: A Psychometric Framework for Large Carnivore Conservation

## Supplementary Online Material

## S1 – Items Found in the Literature

### Coexistence:

Coexistence behavior (Lute & Gore, 2019):

- Attended a legislative hearing or organizational meeting
- Boycotted or avoided buying the products of a company because of their stance on wolf management
- Donated money to a group
- Called or wrote a letter to a legislator
- Educated others
- Managed land to create or conserve wolf habitat
- Read newsletters, magazines or other publications
- Signed a petition
- Volunteered with a group
- Voted for a candidate in an election based at least in part because of his/her stance on wolf management
- Wrote a letter to a newspaper or called in to a news programme

Conservation Behaviors (Lute et al., 2016):

- attended a legislative hearing or organizational meeting
- boycotted or avoided buying the products of a company because of their stance on wolf management
- donated money to a group called or wrote a letter to a legislator
- educated others
- managed land to create or conserve wolf habitat
- read newsletters, magazines or other publications
- signed a petition
- volunteered with a group
- voted for a candidate in an election
- based at least in part because of his/her stance on wolf management
- wrote a letter to a newspaper or called in to a news program

Coexistence behaviors (Lute & Carter, 2020):

- the aggregate mean response concerning the implementation of three self-reported actions (i.e. kept pets inside, removed outside pet food and supervised outdoor pets) in response to actual occurrences of perceived problems with coyotes near the respondent’s home.

Bear attractant securing behavior (Nesbitt et al., 2021):

- Use bear resistant garbage cans
- Have electric fences around attractants (e.g., beehives, chickens, gardens, boneyards)
- Remove fruit from fruit trees
- Use bear resistant pet food/feed storage
- Remove livestock carcasses
- Take down bird feeders during spring and fall months

To quantify willingness to coexistence (Hosaka et al., 2017):

- we asked the respondents to select the most desirable habitat for each of the 29 animals from among the following four locations at different distances from their homes: (1) nowhere, (2) forests and parks distant from one’s home, (3) green spaces near one’s home (within walking distance), or (4) anywhere, including one’s own garden. Again, respondents were not asked to answer these questions if they were unfamiliar with the animal.

Bruskotter et al. (2015):

- Intention to engage in stewardship behavior
  - Write your congressperson in support of further wolf recovery efforts.
  - Contribute to an organization that supports further wolf recovery efforts.
  - Sign a petition in support of further wolf reintroductions.
  - Write a letter to the editor of your local newspaper in support of wolf recovery.
- Intention to engage in intolerant behavior
  - Write your congressperson to oppose further wolf recovery efforts.
  - Contribute to an organization that opposes further wolf recovery efforts.
  - Sign a petition to stop any further wolf reintroductions by the federal government.
  - Write a letter to the editor of your local newspaper opposing wolf recovery.
  - Shoot a wolf if you saw one.
- Prior stewardship behavior (past 6 months)
  - Donated to an organization that supports wolf recovery.
  - Wrote, phoned, or e-mailed my congressperson in support of wolf recovery.
  - Wrote a letter to my newspaper in support of wolf recovery.
- Prior intolerant behavior (past 6 months)
  - Donated to an organization that opposes wolf recovery.
  - Wrote, phoned, or e-mailed my congressperson in opposition to wolf recovery.
  - Wrote a letter to my newspaper in opposition to wolf recovery.

Intention to coexist (Serenari, 2021):

- If red wolves are using private land, tolerating red wolves would be… (unacceptable or acceptable)
- Allowing a USFWS biologist to gradually remove them over time (unacceptable or acceptable)
- Not allowing a USFWS biologist to trap and move them (unacceptable or acceptable)
- Not allowing a USFWS biologist to shoot and kill them (unacceptable or acceptable)

### Cognitive – Perceived Negative Consequences

Cognitive Risk Perception (Lute & Gore, 2019):

- Frequency: Evidence of human–wolf conflict is rare in my community
- Control: I believe that I have control over risks posed by wolves.
- Certainty: If the wolf population increases, human–wolf interactions will increase.
- Trust: I trust wildlife managers to manage wolves appropriately.
- Naturalness: Problems involving wolves are increased by environmental factors.
- Seriousness: The risks posed by wolves are acceptably low.
- Responsiveness: Wildlife managers are responsive to wolf problems.

Perceived cognitive risk of tiger attack (Carter et al., 2012):

- How likely is it that tigers from the nearby forests will attack your pets?
- How likely is it that tigers from the nearby forests will attack your farm animals?
- How likely is it that tigers from the nearby forests will attack someone in your village?
- How likely is it that tigers from the nearby forests will attack you or someone in your family?

Impact beliefs (Frank et al., 2016):

- Coyotes cause significant damages to livestock in NL.
- Coyotes significantly reduce hunting opportunities for big game animals.
- Coyotes are a significant negative impact on the outfitting industry in Newfoundland.

Risk perception (Lute et al., 2020):

- measuring concern using five scenarios (i.e. coyotes attacking children/pets, near home, causing damage to property, spreading rabies).

Perceived risk on people (Amit & Jacobson, 2017):

- Conflicts between people and felids are increasing in your community
- You are worried about walking around your community because of the felids
- You are worried about the safety of children in your community because of the felids

Perceived risk on livestock (Amit & Jacobson, 2017):

- The impact of felid damage to your domestic animals is: none–very high
- The impact of felid damage to your neighbor’s domestic animals is: none–very high
- The risk of any felid damage to your domestic animals in the next 6 months is: none–very high
- The risk of any felid damage to your neighbor’s domestic animals in the next 6 months is: none–very high

Beliefs about the impact of wolves (Jenny Anne Glikman et al., 2012):

- Wolves have a significant impact on big game (e.g., roe deer)
- Wolves have a significant impact on small game (hare)
- Wolves cause abundant damages to livestock

Cognitive component of attitudes: Perceived damages form bears (Jenny A. Glikman et al., 2019):

- Having bears in your region increase tourism
- I would be afraid to hike in the woods if bears were present.
- Bears cause abundant damages to livestock
- Bears cause abundant damages to beehives
- Bears cause abundant damages to orchards and agriculture crops
- Bears should remain completely protected (i.e. it should be illegal to kill them)

Risk from bears (Nesbitt et al., 2021):

- Bears pose a serious risk to belonging on my property (e.g., structures, vehicles)
- Bears pose a serious risk to my family
- Bears pose a serious risk to my pets
- Bears limit my ability to use my property in the way I want
- When bears are attracted to food items on private property, bears’ lives can be threatened

The cognitive factors (i.e., perceived costs), related to the presence of pumas were measured by (Dechner, 2021):

- asking the participants if pumas brought negative (prejudicam) outcomes to them. Responses to these questions were registered separately in a 5-point ordered categorical scale (absolutely no, mostly no, more or less, yes, and very much)

One set of questions was used to generate the variable RISK, which describes the perception respondents have of the threat cougars pose. (Adams Knopff et al., 2016):

- We asked respondents to rank the likelihood of the following scenarios on a five-point scale: (1) encountering a cougar near their home, (2) being attacked by a cougar near their home, (3) their pet being attacked by a cougar near their home, and (4) their livestock being attacked by a cougar.

Risks to humans (Landon et al., 2020):

- Safety of children
- Personal property
- My personal safety

Risks to animals (Landon et al., 2020):

- Deer populations
- Non-hunted wildlife
- Livestock

The cognitive vulnerability model (CVM), using a 6-item version of the instrument (italics indicate reversed coding) (Johansson et al., 2016):

- I think that the movement of wolves is impossible to understand in advance (Move),
- I believe that if I came close to a wolf I would be harmed
- I believe that I would be able to deal effectively with a wolf by myself if encountered (Deal),
- I find wolves to be predictable in their movements (Predict)
- I do not believe wolves could be dangerous to me (Danger)
- If a wolf came nearby I would probably not feel in control (Control).

Vulnerability (Johansson et al., 2017):

- I believe that if I came close to a wolf/bear I would be harmed
- I do not believe wolves/bears could be dangerous to me
- I believe that I would be able to deal effectively with a wolf/bear by myself if encountered
- If a wolf/bear came nearby I would probably not feel in control
- I think that the movement of wolves/bears is impossible to understand in advance
- I find a wolf/bear to be predictable in their movement

Risk perception (Crook, 2019):

- Mountain lions are a threat to people in Santa Cruz County.
- Mountain lions are a threat to livestock and pets in Santa Cruz County.
- I would avoid walking with a child near where a mountain lion was seen in the last two weeks.
- I would avoid walking alone near where a mountain lion was seen in the last two weeks.
- If a mountain lion were seen in my neighborhood, I would alter my behavior in some way.
- A mountain lion would attack a human without being provoked.

Risk (Mcgovern & Kretser, 2015):

- We used a log scale when measuring cognitive risk perceptions. On a scale from a to z, with a being 0 deaths/million people/year due to mountain lion attacks and z being 1 million deaths/million people/year,
- Mountain lions are an unacceptable threat to livestock
- The risks associated with mountain lions living in the Adirondacks are well understood
- If mountain lions lived in the Adirondacks I would:
  - Worry about the safety of my livestock:
  - Worry about children playing outside:
  - Avoid recreating by myself

Beliefs about Negative Outcomes (Slagle et al., 2012):

- Allowing wolf populations to expand into other areas (outside of those areas they currently occupy) would ...
  - result in large numbers of wolf attacks on livestock
  - result in ranchers losing money result in wolf attacks on humans
  - result in wolves wandering into residential areas
  - lead to fewer deer, elk and moose available to hunters
  - result in ranchers killing wolves (dropped from analysis)

Perception of risk (Zajac et al., 2012):

- Black bears will be more of a problem for Ohio in the future
- I am not familiar with the risks posed by black bears
- I am vulnerable to the risks posed by black bears
- I can prevent conflict with black bears by taking precautions around my home. (Reverse coded)
- Conflict with black bears will be reduced as people learn to live with bears. (Reverse coded)
- Encounters with black bears are likely to result in fatal consequences
- I fear having an encounter with a black bear
- All residents are equally exposed to conflicts with black bears in Ohio

Costs (Hiroyasu et al., 2019):

- Grizzly bear reintroduction would lead to an increased role for the federal government.
- Grizzly bear reintroduction would threaten property rights on private lands
- Grizzly bear reintroduction would reduce local control over public lands.
- Grizzly bear reintroduction would negatively impact ranchers.
- Grizzly bear reintroduction would harm agricultural producers.

Personal threats (Hiroyasu et al., 2019):

- whether respondents anticipated needing to change their recreational behavior and
- whether respondents agreed or disagreed that grizzly bear reintroduction would threaten their safety and their livelihood

Risk perception (Heneghan & Morse, 2018):

- Being confronted by a black bear while outdoors
- The cost of residential property damage caused by black bears
- Driving in areas where you may hit a black bear with your vehicle
- The safety of pets that may encounter a black bear
- Conflicts between neighbors over the issue of feeding black bears
- The cost of agricultural damage (e.g. crops, livestock, bee hives) caused by black bears
- The safety of children who may encounter a black bear

Risk perception (Bhatia et al., 2021):

- The population of snow leopards/ wolves is - Increasing | Decreasing | Same | Don’t know
- Do snow leopards/ wolves kill livestock more, or less frequently than they used to?
- Does the snow leopard/ wolf eat only livestock?
- Are you afraid of the snow leopard/ wolf?
- Are snow leopards/ wolves killed as frequently as they used to be?
- If you have seen the snow leopard/ wolf in a non-confrontational situation, did you enjoy the interaction?
  - Were you afraid of the snow leopard/ wolf in the non-confrontational situation?
  - Is it possible to kill a snow leopard/ wolf that kills livestock?
  - Do you think it is possible to protect your livestock better?
  - Does the snow leopard/ wolf benefit the environment or people?

Worry (Morzillo et al., 2010):

- ‘‘I would enjoy having black bears around AND I would not worry about problems they may cause,’’
- ‘‘I would enjoy having black bears around BUT I would worry about the problems they may cause,’’
- ‘‘I would not enjoy having black bears around BUT I would not worry about problems that they may cause,’’
- ‘‘I would not enjoy having black bears around AND I would worry about problems they may cause,’’
- ‘‘I have no particular feelings about black bears regardless of problems caused or not caused by them.’’

Beliefs about the risk of being attacked by a mountain lion were measured by asking participants (Zinn & Pierce, 2002):

- to imagine themselves outside near home and seeing a mountain lion nearby. This scenario was based on actual human-mountain lion encounters that had received media coverage in the study area. Participants rated the likelihood that “the mountain lion would attack you” on a 7point bipolar scale ranging from strongly agree (+3) to strongly disagree (-3).

Cognitive risk judgments about cougars were measured on a risk ladder (Riley & Decker, 2000):

- Respondents were asked to match their perceptions about the safety risks they faced from cougars in terms of probability of human deaths. Probabilities were given on each rung of the ladder that represented a logarithmic scale of potential risk. Respondents were asked to circle a letter along the left side of the ladder that corresponded to their perceptions of the risks from cougars relative to their perceptions of familiar risks given on the right side. Estimates of familiar risks were derived from the National Safety Council (1995), and in the case of air- lines, tractors, automobiles, and motorcycles, rates were specific to Mon- tana.

Perceptions of Outcomes to Reintroducing Wolves (Bright & Manfredo, 1996):

Reintroducing wolves would...

- ... result in large numbers of wolf attacks on livestock.
- ... result in ranchers losing money.
- ... result in wolf attacks on humans.
- ... result in wolves wandering into residential areas.
- ... result in ranchers killing wolves.
- ... lead to large losses in deer and elk populations.

To create a cognitive risk perception index for each HTC incident type (Inskip et al., 2016):

- perceived incident trend and frequency scores for each respondent were combined (Frequency x Trend; range 0–15). Higher index scores represent a higher perceived risk (i.e. an incident type perceived as being common and to be ‘increasing a lot’ received the highest index score (15) while an incident perceived never to occur received the lowest (0) score).

Respondents’ level of concern about tigers (their affective risk perception) was assessed through (Inskip et al., 2016):

- Participatory Risk Mapping. The PRM process comprised two stages: first, respondents were asked to list all of the problems that caused them to worry about their lives, the lives of their family members and/or their livelihoods; and second, they were asked to rank those problems based on the perceived severity of each problem. Standardized severity scores based on the ranks assigned by respondents to their cited problems were then calculated and ranged from 0.2–1, providing an index of concern about tigers. People who did not list tigers as being a problem received a concern score of 0; the remaining concern scores were classified into low ( 0.7), medium (0.7–0.9) and high (>0.9) concern categories based on the distribution of responses.

Perceived risk (Sponarski et al., 2016):

- Because of coyotes, how concerned are you about. . .
  - . . .safety of children
  - . . .property damage caused by a coyote
  - . . .safety of pets
  - . . .contracting rabies

Perceived impact (Marchini & Macdonald, 2012):

- Perceived impact on livestock
  - Damage associated with depredation ever caused to you
  - Damage associated with depredation ever caused to your neighbors
  - Risk of any damage associated with depredation to you in the next 12 months
  - Risk of any damage associated with depredation to your neighbors in the next 12 months
- Perceived impact on human safety
  - Number of people ever hurt by a jaguar in the neighborhood
  - Number of family members ever hurt by a jaguar
  - Risk of you being hurt by a jaguar in the next 12 months
  - Risk of a family member being hurt by a jaguar in the next 12 month

### Cognitive – Perceived Positive Consequences

Beneficial attributes associated with tigers (Carter et al., 2012):

- Do you agree that your village will benefit from more tourism if tigers are in the nearby forests?
- Do you agree that tigers should stay in the nearby forests because they keep the forests healthy?
- Do you agree that tigers are an important part of your culture & should continue roaming the nearby forests?
- Do you agree that tigers were in Chitwan before humans & have the right to live in the nearby forests alongside humans?
- Does it please you just knowing that tigers exist in the nearby forests?
- Do you think that the benefits of living near tigers are greater than, equal to, or less than the risks?

Beliefs (Inskip et al., 2016):

- Tigers benefit people by protecting the Sundarbans
- Tigers benefit people by attracting tourists to the area

Perceptions of Outcomes to Reintroducing Wolves (Bright & Manfredo, 1996):

Reintroducing wolves would...

- ... keep deer and elk populations in balance.
- ... increase tourism in Colorado.
- ... preserve the wolf as a wildlife species.
- ... return the natural environment back to the way it once was.
- ... help people understand the importance of the wilderness.
- ... lead to greater control of rodent populations.

Benefits (Hiroyasu et al., 2019):

- People have a responsibility to ensure the survival of grizzly bears
- Grizzly bear reintroduction would help make California forests healthier.
- Grizzly bear reintroduction would benefit the California economy by increasing tourism.
- Grizzly bear reintroduction would benefit other species.
- Grizzly bear reintroduction would help prevent their extinction.
- Grizzly bear reintroduction would benefit outdoor recreation.
- Grizzly bear reintroduction would benefit urban residents.
- Grizzly bear reintroduction would benefit rural residents.

Beliefs about Positive Outcomes (Slagle et al., 2012):

- Allowing wolf populations to expand into other areas (outside of those areas they currently occupy) would...
  - result in greater control of rodent populations
  - keep deer and elk populations in balance
  - increase tourism in areas where wolves have moved into
  - preserve the wolf as a wildlife species
  - return the natural environment back the way it was

Perception of benefit (Zajac et al., 2012):

- The presence of black bears improves the quality of life in Ohio
- Black bears will provide recreational opportunities for many Ohio residents
- Black bears will improve the ecosystem health of Ohio
- Having black bears in Ohio will be an inconvenience. (Reverse coded)

The cognitive factors (i.e., perceived benefits), related to the presence of pumas were measured by (Dechner, 2021):

- asking the participants if pumas brought positive outcomes to them. Responses to these questions were registered separately in a 5-point ordered categorical scale (absolutely no, mostly no, more or less, yes, and very much)

Recreational Wildlife Experience (Fulton et al., 1996):

- I enjoy watching wildlife when I take a trip outdoors.
- Some of my most memorable outdoor experiences occurred when I saw wildlife I didn't expect to see.
- Some of my most memorable outdoor experiences occurred when I saw wildlife do something I didn't expect.
- One of the reasons I take trips to the outdoors, like camping, hiking or sightseeing, is for the chance to see wildlife.

Benefits (Mcgovern & Kretser, 2015):

- The presence of mountain lions is a sign of a healthy environment
- Mountain lions help maintain deer populations in balance with their habitats
- The presence of mountain lions in the Adirondacks would increase my quality of life
- The presence of mountain lions near my home would increase my overall quality of life
- Mountain lions do not compete with hunters for deer
- Mountain lions should have the right to exist wherever they may occur

Positive belief statements (Sponarski et al., 2016):

- I enjoy seeing coyotes in my current community
- Coyotes are an important part of the ecosystem
- People should learn to live with coyotes
- Seeing coyotes makes me feel connected to nature

### Cognitive – Ethical Considerations

Beneficial attributes associated with tigers (Carter et al., 2012):

- Do you agree that tigers are an important part of your culture & should continue roaming the nearby forests?
- Do you agree that tigers were in Chitwan before humans & have the right to live in the nearby forests alongside humans?
- Does it please you just knowing that tigers exist in the nearby forests?
- Do you think that the benefits of living near tigers are greater than, equal to, or less than the risks?

Existence beliefs (Frank et al., 2016):

- It is important to maintain populations of coyotes in Newfoundland for future generations.
- Whether or not I would get to see coyote it is important to me they exist in Newfoundland.

Beliefs (Inskip et al., 2016):

- Tigers are good animals Tigers should be protected

Symbolic Existence Beliefs About Wolves (Bright & Manfredo, 1996):

- It is important that Colorado always have an abundant wolf population.
- Whether or not I would get to see a wolf, it is important to me that they exist in Colorado.
- We should be sure that future generations of Coloradoans have an abundant wolf population.
- It would be important to me to know that there are healthy populations of wolves in Colorado.
- It is important to maintain wolf populations in Colorado so future generations can enjoy them.

Bequest and Existence (Fulton et al., 1996):

- It is important that Colorado always have abundant fish and wildlife.
- Whether or not I get out to see wildlife as much as I'd like, ifs important to know that they exist in Colorado.
- We should be sure future generations of Colorado will have an abundance of fish and wildlife.
- It's important to me to know that there are healthy populations of wildlife in Colorado.
- It is important to maintain fish and wildlife so that future generations can enjoy them.

Residential Wildlife Experience (Fulton et al., 1996):

- I enjoy seeing birds around my home.
- I notice the birds and wildlife around me every day.
- Having wildlife around my home is important to me.
- I'm interested in making the area around my home attractive to birds and wildlife.
- An important part of my community is the wildlife I see there from time to time.

Wildlife Education (Fulton et al., 1996):

- I enjoy learning about wildlife.
- It is important that all Colorado residents have a chance to learn about wildlife in the state.
- It is important that we learn as much as we can about wildlife.

Normative beliefs about mountain lion existence (Davenport et al., 2010):

- Mountain lions should have the right to exist wherever they may occur

The Animal Rights and Scale (Wuensch et al., 2002):

- 1. Humans have no right to displace wild animals by converting wilderness areas into farmlands, cities, and other things designed for people.
- 2. Animal research cannot be justified and should be stopped.
- 3. It is morally wrong to drink milk and eat eggs.
- 4. A human has no right to use a horse as a means of transportation (riding) or entertainment (racing).
- 5. It is wrong to wear leather jackets and pants.
- 6. Most medical research done on animals is unnecessary and invalid.
- 7. I have seriously considered becoming a vegetarian in an effort to save animal lives.
- 8. Pet owners are responsible for preventing their pets from killing other animals, such as cats killing mice or snakes eating live mice.
- 9. We need more regulations governing the use of animals in research.
- 10. It is morally wrong to eat beef and other “red” meat.
- 11. Insect pests (mosquitoes, cockroaches, flies, etc.) should be safely removed from the house rather than killed.
- 12. Animals should be granted the same rights as humans.
- 13. It is wrong to wear leather belts and shoes.
- 14. I would rather see humans die or suffer from disease than to see animals used in research.
- 15. Having extended basic rights to minorities and women, it is now time to extend them also to animals.
- 16. God put animals on Earth for man to use.
- 17. There are plenty of viable alternatives to the use of animals in biomedical and behavioral research.
- 18. Research on animals has little or no bearing on problems confronting people.
- 19. New surgical procedures and experimental drugs should be tested on animals before they are used on people.
- 20. I am very concerned about pain and suffering in animals.
- 21. Since many important questions cannot be answered by doing experiments on people, we are left with no alternatives but to do animal research.
- 22. It is a violation of an animal’s rights to be held captive as a pet by a human.
- 23. It is wrong to wear animal fur (such as mink coats).
- 24. It is appropriate for humans to kill animals that destroy human property, for example, rats, mice, and pigeons.
- 25. Most cosmetics research done on animals is unnecessary and invalid.
- 26. It is morally wrong to eat chicken and fish.
- 27. Most psychological research done on animals is unnecessary and invalid.
- 28. Hunters play an important role in regulating the size of deer populations.

Wildlife Rights (Fulton et al., 1996):

- The rights of wildlife are more important than human use of wildlife.
- Animals should have rights similar to the rights of humans.
- I object to hunting because it violates the rights of an individual animal to exist

## S2 – Expert Review of the Pool of Items

### Willingness to Coexist – Private Sphere

| Item | Relevancy | Universality | Clarity |
| --- | --- | --- | --- |
| 1. I am willing to alter the activities that I do outdoor in [carnivore species'] habitat to accommodate living with them in my area. | 4.38 | 3.62 | 3.46 |
| 2. I am willing to take measures to protect myself, my family, and my properties from [carnivore species] to facilitate living with them in my area. | 4.33 | 3.58 | 3.42 |
| 3. I am willing to take measures to reduce potential risks that [carnivore species] may impose to my life or livelihood to facilitate living with them in my area. | 4.31 | 3.62 | 3.46 |
| 4. I am willing to tolerate a certain extent of damage to my property as a price for living with [carnivore species] in my area. | 4.62 | 4.00 | 4.00 |
| 5. I am willing to alter my business practices to accommodate living with [carnivore species] in my area. | 4.31 | 3.38 | 3.54 |
| 6. I am willing to support activities that reduce threats to [carnivore species] populations in my area. | 4.62 | 4.23 | 3.75 |
| 7. I am willing to support products and businesses that promote living with [carnivore species] in my area. | 4.31 | 3.69 | 3.69 |
| 8. I am willing to educate myself to facilitate living with [carnivore species] in my area. | 4.58 | 4.50 | 4.08 |

### Willingness to Coexist – Private Sphere

| Item | Relevancy | Universality | Clarity |
| --- | --- | --- | --- |
| 1. I am willing to support public initiatives that facilitate living with [carnivore species] in my area. | 4.42 | 3.83 | 3.50 |
| 2. I am willing to try to encourage my peers to support living with [carnivore species] in my area. | 4.67 | 4.73 | 4.27 |
| 3. I am willing to vote for initiatives that foster living with [carnivore species] in my area. | 4.50 | 3.50 | 3.83 |
| 4. I am willing to contribute resources (e.g., time, money) to groups, organizations, or initiatives that promote [carnivore species’] presence in my area. | 4.67 | 4.25 | 4.17 |
| 5. I am willing to try to convince the authorities to support living with [carnivore species] in my area. | 3.73 | 3.91 | 3.45 |
| 6. I am willing to try to educate my peers to facilitate living with [carnivore species] in my area. | 4.50 | 4.50 | 4.09 |

### Perceived Negative Consequences

| Item | Relevancy | Universality | Clarity |
| --- | --- | --- | --- |
| 1. Living with [carnivore species] in my area benefits me. | 4.92 | 4.67 | 4.50 |
| 2. Having [carnivore species] in my area restores the ecosystem balance. | 4.73 | 4.18 | 4.00 |
| 3. [Carnivore species] benefit people by attracting tourists to my area. | 4.83 | 4.25 | 4.33 |
| 4. [Carnivore species] will improve the ecosystem health of my area. | 4.33 | 4.00 | 3.83 |
| 5. [Carnivore species’] presence in my area lead to greater control of pest populations. | 4.33 | 3.83 | 4.00 |
| 6. [Carnivore species] will provide recreational opportunities for many residents in my area. | 4.09 | 3.36 | 3.45 |
| 7. [Carnivore species’] presence improves the quality of life in my area. | 4.33 | 4.25 | 4.00 |
| 8. I enjoy watching [carnivore species] when I take a trip outdoors. | 4.50 | 4.17 | 4.50 |
| 9. Having [carnivore species] in my area benefits other species. | 4.73 | 4.55 | 4.36 |

### Perceived Positive Consequences

| Item | Relevancy | Universality | Clarity |
| --- | --- | --- | --- |
| 1. Living with [carnivore species] in my area threatens my personal safety. | 4.67 | 4.58 | 4.75 |
| 2. Living with [carnivore species] in my area threatens my family’s safety. | 4.67 | 4.50 | 4.67 |
| 3. Living with [carnivore species] in my area threatens the safety of my pets. | 4.75 | 3.75 | 4.33 |
| 4. Living with [carnivore species] in my area threatens my livelihood. | 4.92 | 4.42 | 4.75 |
| 5. Living with [carnivore species] in my area threatens the safety of my community. | 4.33 | 4.17 | 4.00 |
| 6. [Carnivore species] significantly reduce hunting opportunities for game animals in my area. | 4.50 | 3.50 | 3.92 |
| 7. [Carnivore species] are a threat to livestock and agriculture products in my area. | 4.58 | 4.08 | 4.25 |
| 8. It would be dangerous to walk in the open spaces in my area if [carnivore species] were present. | 4.45 | 4.00 | 3.82 |
| 9. [Carnivore species] pose a serious risk to belongings on my property (e.g., structures, vehicles). | 4.27 | 3.73 | 3.64 |
| 10. [Carnivore species] limit my ability to use my property in the way I want. | 4.67 | 3.92 | 4.33 |
| 11. If a [carnivore species] were seen in my neighborhood, I would alter my behavior in some way. | 4.42 | 3.92 | 3.67 |
| 12. A [carnivore species] would attack a human without being provoked. | 4.45 | 4.36 | 4.45 |

### Ethical Considerations

| Item | Relevancy | Universality | Clarity |
| --- | --- | --- | --- |
| 1. It is important that my area always has a healthy [carnivore species] population. | 4.50 | 4.42 | 3.92 |
| 2. [Carnivore species] have the right to live wherever they may occur. | 4.00 | 3.92 | 3.83 |
| 3. [Carnivore species] have the right to live wherever they were once common. | 4.25 | 3.92 | 3.58 |
| 4. It is important to maintain populations of [carnivore species] in my area for future generations. | 4.83 | 4.67 | 4.50 |
| 5. Whether or not I would get to see [carnivore species], it is important to me they exist in my area. | 4.83 | 4.58 | 4.83 |
| 6. I enjoy seeing [carnivore species] in my area. | 4.67 | 4.58 | 4.75 |
| 7. [Carnivore species] are an important part of the ecosystem in my area. | 4.00 | 3.92 | 4.00 |
| 8. Seeing [carnivore species] in my area makes me feel connected to nature. | 4.33 | 4.17 | 4.42 |
| 9. It is important that my area always has an abundant [carnivore species] population. | 4.00 | 3.92 | 3.92 |
| 10. It would be important to me to know that there are healthy populations of [carnivore species] in my area. | 4.00 | 3.92 | 3.73 |
| 11. [Carnivore species] are an important part of my culture and should continue roaming in my area. | 4.55 | 4.00 | 4.18 |
| 12. Having [carnivore species] in my area improves the sense of wilderness. | 3.73 | 3.36 | 3.36 |
| 13. Having [carnivore species] in my area preserve [carnivore species] as a wildlife species. | 3.20 | 3.11 | 2.50 |

## S3 – Model goodness of fit statistics used for testing dimensionality

Figure 1 – Hypothesized measurement models to test the dimensionality of willingness to coexist (top) and beliefs about coexistence (bottom).

### Wolf sample:

| Model | Overall fit statistics | | | |
| --- | --- | --- | --- | --- |
|  | χ^2^ (df) | CFI | TLI | RMSEA [90% CI] |
| WCoEx | | | | |
| 1-Factor | 316.515 (72) | 0.961 | 0.943 | 0.089 [0.079; 0.099] |
| 2-Factor | 273.151 (71) | 0.967 | 0.952 | 0.081 [0.071; 0.091] |
| Second-order | 273.151 (71) | 0.967 | 0.952 | 0.081 [0.071; 0.091] |
| Beliefs about coexistence | | | | |
| 1-Factor | 457.801 (113) | 0.948 | 0.921 | 0.084 [0.076; 0.092] |
| 3-Factor | 182.983 (110) | 0.989 | 0.983 | 0.039 [0.029; 0.049] |
| Second-order | 197.731 (111) | 0.987 | 0.980 | 0.042 [0.033; 0.052] |

### Mountain lion sample:

| Model | Overall fit statistics | | | |
| --- | --- | --- | --- | --- |
|  | χ^2^ (df) | CFI | TLI | RMSEA [90% CI] |
| WCoEx | | | | |
| 1-Factor | 377.118 (72) | 0.949 | 0.926 | 0.098 [0.088; 0.108] |
| 2-Factor | 254.025 (71) | 0.970 | 0.955 | 0.076 [0.066; 0.087] |
| Second-order | 254.025 (71) | 0.970 | 0.955 | 0.076 [0.066; 0.087] |
| Beliefs about coexistence | | | | |
| 1-Factor | 629.006 (113) | 0.919 | 0.877 | 0.102 [0.094; 0.110] |
| 3-Factor | 244.053 (110) | 0.979 | 0.967 | 0.053 [0.044; 0.061] |
| Second-order | 245.918 (111) | 0.979 | 0.967 | 0.052 [0.044; 0.061] |

### Black bear sample:

| Model | Overall fit statistics | | | |
| --- | --- | --- | --- | --- |
|  | χ^2^ (df) | CFI | TLI | RMSEA [90% CI] |
| WCoEx | | | | |
| 1-Factor | 414.962 (72) | 0.940 | 0.913 | 0.105 [0.095; 0.115] |
| 2-Factor | 288.795 (71) | 0.962 | 0.944 | 0.084 [0.074; 0.095] |
| Second-order | 288.795 (71) | 0.962 | 0.944 | 0.084 [0.074; 0.095] |
| Beliefs about coexistence | | | | |
| 1-Factor | 613.473 (113) | 0.901 | 0.850 | 0.101 [0.093; 0.109] |
| 3-Factor | 199.816 (110) | 0.982 | 0.972 | 0.043 [0.034; 0.053] |
| Second-order | 206.026 (111) | 0.981 | 0.971 | 0.045 [0.035; 0.054] |

## S4 – Measurement invariance testing results based on the two-factor model for WCoEx and the three-factor model for beliefs about coexistence

| Model | χ^2^ (df) | CFI | TLI | RMSEA [90% CI] | ΔCFI |
| --- | --- | --- | --- | --- | --- |
| WCoEx | | | | | |
| Unconstrained  (configural invariance) | 815.972 (213) | 0.966 | 0.950 | 0.047 [0.043; 0.050] | - |
| Measurement weights  (factorial invariance) | 876.497 (237) | 0.964 | 0.953 | 0.045 [0.042; 0.049] | -0.002 |
| Measurement intercepts  (scalar invariance) | 1006.451 (265) | 0.959 | 0.951 | 0.046 [0.043; 0.049] | -0.007 |
| Beliefs about coexistence | | | | | |
| Unconstrained  (configural invariance) | 932.300 (409) | 0.971 | 0.964 | 0.031 [0.029; 0.034] | - |
| Measurement weights  (factorial invariance) | 971.957 (424) | 0.970 | 0.963 | 0.031 [0.029; 0.034] | -0.001 |
| Measurement intercepts  (scalar invariance) | 1168.248 (442) | 0.960 | 0.953 | 0.035 [0.033; 0.038] | -0.011 |

## S5 – Descriptive Statistics and Scale Reliability of Constructs Used for Predictive and Convergent Validity Testing

### Wolf sample:

| Construct / item | *M* | *SD* | ω |
| --- | --- | --- | --- |
| Trust in state wildlife agency^a^ |  |  |  |
| Overall, I trust CPW | 4.92 | 1.58 | 0.94 |
| I feel CPW shares similar values as me with respect to wildlife management | 4.84 | 1.59 |  |
| I trust CPW to provide the best available information on wolf reintroduction/management | 5.02 | 1.67 |  |
| I trust CPW to make decisions about wolf reintroduction/management that are in the best interest of wolves and Coloradans | 4.92 | 1.72 |  |
| Wildlife value orientations - mutualism^a^ |  |  |  |
| Animals should have rights similar to the rights of humans | 3.69 | 1.73 | 0.82 |
| I view all living things as part of one big family | 4.63 | 1.65 |  |
| Wildlife are like my family and I want to protect them | 4.39 | 1.54 |  |
| Wildlife value orientations - domination^a^ |  |  |  |
| Humans should manage wildlife populations so that humans benefit | 3.87 | 1.52 | 0.77 |
| The needs of humans should take priority over wildlife protection | 3.88 | 1.66 |  |
| Wildlife are on earth primarily for people to use. | 2.71 | 1.58 |  |
| Please indicate the degree to which you disapprove or approve of reintroducing wolves into Colorado. | 4.46 | 2.24 | N.A. |
| Willingness to pay for wolf^b^ | -21.00 | 32.29 | N.A. |
| ^a^ Coded from 1 to 7, ‘Strongly disagree’ to ‘Strongly agree’  ^b^ Coded from 1 to 7, ‘Strongly disapprove’ to ‘Strongly approve’ | | | |

### Mountain lion sample:

| Construct / item | *M* | *SD* | ω |
| --- | --- | --- | --- |
| Wildlife value orientations – mutualism^a^ |  |  |  |
| Animals should have rights similar to the rights of humans | 2.96 | 1.47 | 0.84 |
| I view all living things as part of one big family | 3.62 | 1.28 |  |
| Wildlife are like my family and I want to protect them | 3.35 | 1.32 |  |
| Wildlife value orientations - domination^a^ |  |  |  |
| Humans should manage wildlife populations so that humans benefit | 2.80 | 1.28 | 0.74 |
| The needs of humans should take priority over wildlife protection | 2.70 | 1.27 |  |
| Wildlife are on earth primarily for people to use | 1.98 | 1.98 |  |
| Preventive measures^b^ |  |  |  |
| Keeping pets indoors | 4.10 | 2.17 | 0.88 |
| Providing pets with a covered outdoor run | 4.00 | 2.14 |  |
| Keeping pets on leash while recreating in mountain lion habitat | 5.25 | 1.95 |  |
| Feeding pets indoors | 5.47 | 1.91 |  |
| Avoid attracting mountain lion prey to house yards (for example, deer, raccoons) | 5.18 | 1.93 |  |
| Exercising caution when recreating in mountain lion habitat | 5.67 | 1.62 |  |
| Safeguarding livestock | 5.15 | 1.83 |  |
| Over the next 5 years, would you like to see the mountain lion population in Colorado^c^ | 4.29 | 1.39 | N.A. |
| ^a^ Coded from 1 to 5, ‘Strongly disagree’ to ‘Strongly agree’  ^b^ Coded from 1 to 6, ‘Not at all willing’ to ‘Extremely willing’  ^c^ Coded from 1 to 7, ‘Decreases a great deal’ to ‘Increases a great deal’ | | | |

### Black bear sample:

| Construct / item | *M* | *SD* | ω |
| --- | --- | --- | --- |
| Wildlife value orientations – mutualism |  |  |  |
| Animals should have rights similar to the rights of humans | 2.97 | 1.44 | 0.84 |
| I view all living things as part of one big family | 3.67 | 1.29 |  |
| Wildlife are like my family and I want to protect them | 3.50 | 1.23 |  |
| Wildlife value orientations - domination |  |  |  |
| Humans should manage wildlife populations so that humans benefit | 2.89 | 1.29 | 0.73 |
| The needs of humans should take priority over wildlife protection | 2.73 | 1.31 |  |
| Wildlife are on earth primarily for people to use | 2.03 | 1.22 |  |
| Preventive measures |  |  |  |
| Keeping pets inside | 4.18 | 2.11 | 0.91 |
| Providing pets with a covered outdoor run | 4.16 | 2.18 |  |
| Keeping pets on leash while recreating in black bear habitat | 5.40 | 1.92 |  |
| Feeding pets indoors | 5.76 | 1.75 |  |
| Keeping garbage secured until morning of pickup day, rather than putting it out the night before | 5.83 | 1.69 |  |
| Not having composters, gardens, or fruit trees | 3.12 | 2.11 |  |
| Removing bird, squirrel, and other wildlife feeders | 4.34 | 2.19 |  |
| Using a wildlife-resistant garbage container or dumpster | 5.95 | 1.60 |  |
| Exercising caution when recreating in black bear habitat | 6.01 | 1.48 |  |
| Safeguarding livestock | 5.28 | 1.79 |  |
| Keeping doors and windows of houses and vehicles locked | 5.56 | 1.83 |  |
| Over the next 5 years, would you like to see the mountain lion population in Colorado | 4.48 | 1.21 | N.A. |
| ^a^ Coded from 1 to 5, ‘Strongly disagree’ to ‘Strongly agree’  ^b^ Coded from 1 to 6, ‘Not at all willing’ to ‘Extremely willing’  ^c^ Coded from 1 to 7, ‘Decreases a great deal’ to ‘Increases a great deal’ | | | |

## S6 – Item factor loadings and scale reliability coefficients of the final scale items

### Wolf sample:

| Construct / item | λ | *CR* | ω |
| --- | --- | --- | --- |
| WCoEx–Individual |  | 0.896 | 0.903 |
| CoEx-Ind1 - Change my behavior to reduce potential conflicts between people and wolves. | 0.84 |  |  |
| CoEx-Ind2 - Accept some negative impact from wolves on my livelihood or source of income. | 0.74 |  |  |
| CoEx-Ind3 - Learn about ways to reduce potential conflicts between people and wolves. | 0.79 |  |  |
| CoEx-Ind4 - Oppose killing a wolf unless my personal or family’s safety is at risk. | 0.67 |  |  |
| CoEx-Ind5 - Oppose killing a wolf unless my animals (pets, livestock, etc.) are at risk. | 0.59 |  |  |
| CoEx-Ind6 - Oppose killing a wolf unless my property (home, vehicle, etc.) is at risk. | 0.42 |  |  |
| CoEx-Ind7 - Find ways to protect my livestock or property from wolves without harming the wolf. | 0.81 |  |  |
| CoEx-Ind8 - Find ways to protect myself, my family, or my pets from wolves without harming the wolf. | 0.80 |  |  |
| WCoEx–Collective |  | 0.963 | 0.963 |
| CoEx-Col1 - Support policies and management actions to conserve wolves. | 0.93 |  |  |
| CoEx-Col2 - Support the efforts of wildlife management authorities working to help people share the landscape with wolves. | 0.93 |  |  |
| CoEx-Col3 - Encourage wildlife management authorities to promote ways to share the landscape with wolves. | 0.92 |  |  |
| CoEx-Col4 - Encourage my friends and family to promote ways to share the landscape with wolves. | 0.91 |  |  |
| CoEx-Col5 - Work with my community to promote ways to share the landscape with wolves. | 0.90 |  |  |
| CoEx-Col6 - Contribute time or money to programs that help people share the landscape with wolves. | 0.82 |  |  |
| Perceived positive consequences |  | 0.932 | 0.947 |
| *Having wolves* *in the area where I live would…* |  |  |  |
| PPO1 - benefit me. | 0.79 |  |  |
| PPO2 - be good for the overall health of the environment. | 0.89 |  |  |
| PPO3 - benefit my community. | 0.87 |  |  |
| PPO4 - improve my quality of life. | 0.80 |  |  |
| PPO5 - benefit other animals and plants. | 0.89 |  |  |
| PPO6 - be an important part of my culture. | 0.77 |  |  |
| Perceived negative consequences |  | 0.887 | 0.905 |
| *Having wolves* *in the area where I live would…* |  |  |  |
| PNO1 - threaten my personal or family’s safety. | 0.79 |  |  |
| PNO2 - threaten the safety of my animals (pets, livestock, etc.). | 0.75 |  |  |
| PNO3 - threaten my property, livelihood, or income. | 0.76 |  |  |
| PNO4 - limit my ability to use my land the way I want. | 0.75 |  |  |
| PNO5 - threaten livestock or agricultural production in my community. | 0.65 |  |  |
| PNO6 - threaten local outdoor recreation opportunities (recreational hunting, bird watching, hiking, etc.). | 0.80 |  |  |
| Ethical considerations |  | 0.894 | 0.916 |
| ETH1 - It would be important to maintain wolves in my area for future human generations. | 0.94 |  |  |
| ETH2 - Whether or not I get to see wolves, it would be important to know that they exist in my area. | 0.75 |  |  |
| ETH3 - Wolves deserve to be conserved for their own value. | 0.78 |  |  |
| ETH4 - Individual wolves should not be harmed by humans without cause. | 0.62 |  |  |
| ETH5 - Wolves have value, above and beyond their use to humans. | 0.74 |  |  |
| ETH6 - Wolves should exist whether they serve human needs or not. | 0.74 |  |  |
| *Note*. λ = standardized factor loading; *CR* = composite reliability; ω = McDonald’s omega; WCoEx items were rated on a five-point scale, ranging from "Not at all willing" (0) to "Extremely willing" (4). Beliefs about coexistence items used a five-point Likert-type scale from "Strongly disagree" (-2) to "Strongly agree" (+2). | | | |

### Mountain lion sample:

| Construct / item | λ | *CR* | ω |
| --- | --- | --- | --- |
| WCoEx–Individual |  | 0.902 | 0.918 |
| CoEx-Ind1 - Change my behavior to reduce potential conflicts between people and mountain lions. | 0.80 |  |  |
| CoEx-Ind2 - Accept some negative impact from mountain lions on my livelihood or source of income. | 0.68 |  |  |
| CoEx-Ind3 - Learn about ways to reduce potential conflicts between people and mountain lions. | 0.77 |  |  |
| CoEx-Ind4 - Oppose killing a mountain lion unless my personal or family’s safety is at risk. | 0.67 |  |  |
| CoEx-Ind5 - Oppose killing a mountain lion unless my animals (pets, livestock, etc.) are at risk. | 0.68 |  |  |
| CoEx-Ind6 - Oppose killing a mountain lion unless my property (home, vehicle, etc.) is at risk. | 0.63 |  |  |
| CoEx-Ind7 - Find ways to protect my livestock or property from mountain lions without harming the mountain lion. | 0.79 |  |  |
| CoEx-Ind8 - Find ways to protect myself, my family, or my pets from mountain lions without harming the mountain lion. | 0.82 |  |  |
| WCoEx–Collective |  | 0.959 | 0.955 |
| CoEx-Col1 - Support policies and management actions to conserve mountain lions. | 0.89 |  |  |
| CoEx-Col2 - Support the efforts of wildlife management authorities working to help people share the landscape with mountain lions. | 0.94 |  |  |
| CoEx-Col3 - Encourage wildlife management authorities to promote ways to share the landscape with mountain lions. | 0.94 |  |  |
| CoEx-Col4 - Encourage my friends and family to promote ways to share the landscape with mountain lions. | 0.93 |  |  |
| CoEx-Col5 - Work with my community to promote ways to share the landscape with mountain lions. | 0.88 |  |  |
| CoEx-Col6 - Contribute time or money to programs that help people share the landscape with mountain lions. | 0.73 |  |  |
| Perceived positive consequences |  | 0.926 | 0.941 |
| *Having mountain lions* *in the area where I live would…* |  |  |  |
| PPO1 - benefit me. | 0.79 |  |  |
| PPO2 - be good for the overall health of the environment. | 0.89 |  |  |
| PPO3 - benefit my community. | 0.85 |  |  |
| PPO4 - improve my quality of life. | 0.78 |  |  |
| PPO5 - benefit other animals and plants. | 0.83 |  |  |
| PPO6 - be an important part of my culture. | 0.80 |  |  |
| Perceived negative consequences |  | 0.909 | 0.916 |
| *Having mountain lions* *in the area where I live would…* |  |  |  |
| PNO1 - threaten my personal or family’s safety. | 0.83 |  |  |
| PNO2 - threaten the safety of my animals (pets, livestock, etc.). | 0.72 |  |  |
| PNO3 - threaten my property, livelihood, or income. | 0.80 |  |  |
| PNO4 - limit my ability to use my land the way I want. | 0.80 |  |  |
| PNO5 - threaten livestock or agricultural production in my community. | 0.76 |  |  |
| PNO6 - threaten local outdoor recreation opportunities (recreational hunting, bird watching, hiking, etc.). | 0.84 |  |  |
| Ethical considerations |  | 0.885 | 0.904 |
| ETH1 - It would be important to maintain wolves/mountain lions/black bears in my area for future human generations. | 0.88 |  |  |
| ETH2 - Whether or not I get to see wolves/mountain lions/black bears, it would be important to know that they exist in my area. | 0.71 |  |  |
| ETH3 - Wolves/mountain lions/black bears deserve to be conserved for their own value. | 0.77 |  |  |
| ETH4 - Individual wolves/mountain lions/black bears should not be harmed by humans without cause. | 0.62 |  |  |
| ETH5 - Wolves/mountain lions/black bears have value, above and beyond their use to humans. | 0.75 |  |  |
| ETH6 - Wolves/mountain lions/black bears should exist whether they serve human needs or not. | 0.75 |  |  |
| *Note*. λ = standardized factor loading; *CR* = composite reliability; ω = McDonald’s omega; WCoEx items were rated on a five-point scale, ranging from "Not at all willing" (0) to "Extremely willing" (4). Beliefs about coexistence items used a five-point Likert-type scale from "Strongly disagree" (-2) to "Strongly agree" (+2). | | | |

### Black bear sample:

| Construct / item | λ | *CR* | ω |
| --- | --- | --- | --- |
| WCoEx–Individual |  | 0.893 | 0.909 |
| CoEx-Ind1 - Change my behavior to reduce potential conflicts between people and black bears. | 0.75 |  |  |
| CoEx-Ind2 - Accept some negative impact from black bears on my livelihood or source of income. | 0.65 |  |  |
| CoEx-Ind3 - Learn about ways to reduce potential conflicts between people and black bears. | 0.79 |  |  |
| CoEx-Ind4 - Oppose killing a black bear unless my personal or family’s safety is at risk. | 0.60 |  |  |
| CoEx-Ind5 - Oppose killing a black bear unless my animals (pets, livestock, etc.) are at risk. | 0.57 |  |  |
| CoEx-Ind6 - Oppose killing a black bear unless my property (home, vehicle, etc.) is at risk. | 0.61 |  |  |
| CoEx-Ind7 - Find ways to protect my livestock or property from black bears without harming the black bear. | 0.84 |  |  |
| CoEx-Ind8 - Find ways to protect myself, my family, or my pets from black bears without harming the black bear. | 0.87 |  |  |
| WCoEx–Collective |  | 0.952 | 0.949 |
| CoEx-Col1 - Support policies and management actions to conserve black bears. | 0.87 |  |  |
| CoEx-Col2 - Support the efforts of wildlife management authorities working to help people share the landscape with black bears. | 0.93 |  |  |
| CoEx-Col3 - Encourage wildlife management authorities to promote ways to share the landscape with black bears. | 0.94 |  |  |
| CoEx-Col4 - Encourage my friends and family to promote ways to share the landscape with black bears. | 0.93 |  |  |
| CoEx-Col5 - Work with my community to promote ways to share the landscape with black bears. | 0.86 |  |  |
| CoEx-Col6 - Contribute time or money to programs that help people share the landscape with black bears. | 0.73 |  |  |
| Perceived positive consequences |  | 0.907 | 0.918 |
| *Having black bears* *in the area where I live would…* |  |  |  |
| PPO1 - benefit me. | 0.78 |  |  |
| PPO2 - be good for the overall health of the environment. | 0.82 |  |  |
| PPO3 - benefit my community. | 0.81 |  |  |
| PPO4 - improve my quality of life. | 0.77 |  |  |
| PPO5 - benefit other animals and plants. | 0.79 |  |  |
| PPO6 - be an important part of my culture. | 0.75 |  |  |
| Perceived negative consequences |  | 0.900 | 0.907 |
| *Having black bears* *in the area where I live would…* |  |  |  |
| PNO1 - threaten my personal or family’s safety. | 0.83 |  |  |
| PNO2 - threaten the safety of my animals (pets, livestock, etc.). | 0.75 |  |  |
| PNO3 - threaten my property, livelihood, or income. | 0.82 |  |  |
| PNO4 - limit my ability to use my land the way I want. | 0.74 |  |  |
| PNO5 - threaten livestock or agricultural production in my community. | 0.75 |  |  |
| PNO6 - threaten local outdoor recreation opportunities (recreational hunting, bird watching, hiking, etc.). | 0.75 |  |  |
| Ethical considerations |  | 0.848 | 0.865 |
| ETH1 - It would be important to maintain wolves/mountain lions/black bears in my area for future human generations. | 0.86 |  |  |
| ETH2 - Whether or not I get to see wolves/mountain lions/black bears, it would be important to know that they exist in my area. | 0.85 |  |  |
| ETH3 - Wolves/mountain lions/black bears deserve to be conserved for their own value. | 0.75 |  |  |
| ETH4 - Individual wolves/mountain lions/black bears should not be harmed by humans without cause. | 0.39 |  |  |
| ETH5 - Wolves/mountain lions/black bears have value, above and beyond their use to humans. | 0.58 |  |  |
| ETH6 - Wolves/mountain lions/black bears should exist whether they serve human needs or not. | 0.68 |  |  |
| *Note*. λ = standardized factor loading; *CR* = composite reliability; ω = McDonald’s omega; WCoEx items were rated on a five-point scale, ranging from "Not at all willing" (0) to "Extremely willing" (4). Beliefs about coexistence items used a five-point Likert-type scale from "Strongly disagree" (-2) to "Strongly agree" (+2). | | | |

## S7 – Evidence for convergent and discriminant validity

### Wolf sample:

|  | AVE | 1 | 2 | 3 |
| --- | --- | --- | --- | --- |
| WCoEx | | | | |
| 1. Individual | 0.527 | - | Δχ^2^ = 27.441 |  |
| 2. Collective | 0.812 | *r* = 0.945 | - |  |
| Beliefs about coexistence | | | | |
| 1. Perceived positive consequences | 0.697 | - | Δχ^2^ = 3250.109 | Δχ^2^ = 46.414 |
| 2. Perceived negative consequences | 0.567 | *r* = -0.740 | - | Δχ^2^ = 2514.707 |
| 3. Ethical considerations | 0.589 | *r* = 0.873 | *r* = -0.686 | - |
| *Note*. AVE = Average Variance Extracted; Lower diagonal values represent correlations (*r*) obtained from the measurement model estimations; Upper diagonal values represent chi-square differences (Δχ^2^) with Δdf = 1 for the latent factor correlation constraint test (all tests were statistically significant at the < 0.001 level). | | | | |

### Mountain lion sample:

|  | AVE | 1 | 2 | 3 |
| --- | --- | --- | --- | --- |
| WCoEx | | | | |
| 1. Individual | 0.536 | - | Δχ^2^ = 55.806 |  |
| 2. Collective | 0.798 | *r* = 0.884 | - |  |
| Beliefs about coexistence | | | | |
| 1. Perceived positive consequences | 0.675 | - | Δχ^2^ = 2781.138 | Δχ^2^ = 61.273 |
| 2. Perceived negative consequences | 0.626 | *r* = -0.694 | - | Δχ^2^ = 2200.567 |
| 3. Ethical considerations | 0.564 | *r* = 0.814 | *r* = -0.647 | - |
| *Note*. AVE = Average Variance Extracted; Lower diagonal values represent correlations (*r*) obtained from the measurement model estimations; Upper diagonal values represent chi-square differences (Δχ^2^) with Δdf = 1 for the latent factor correlation constraint test (all tests were statistically significant at the < 0.001 level). | | | | |

### Black bear sample:

|  | AVE | 1 | 2 | 3 |
| --- | --- | --- | --- | --- |
| WCoEx | | | | |
| 1. Individual | 0.515 | - | Δχ^2^ = 56.583 |  |
| 2. Collective | 0.768 | *r* = 0.854 | - |  |
| Beliefs about coexistence | | | | |
| 1. Perceived positive consequences | 0.620 | - | Δχ^2^ = 2111.620 | Δχ^2^ = 75.827 |
| 2. Perceived negative consequences | 0.599 | *r* = -0.656 | - | Δχ^2^ = 1126.932 |
| 3. Ethical considerations | 0.495 | *r* = 0.697 | *r* = -0.497 | - |
| *Note*. AVE = Average Variance Extracted; Lower diagonal values represent correlations (*r*) obtained from the measurement model estimations; Upper diagonal values represent chi-square differences (Δχ^2^) with Δdf = 1 for the latent factor correlation constraint test (all tests were statistically significant at the < 0.001 level). | | | | |

## S8 – Mplus Syntaxes Used for SEM Analysis

Note: Example syntaxes are provided.

### Dimensionality Tests

Second-order factor model of willingness to coexist

DATA: FILE = CoEx_Wolf.csv;

VARIABLE:

NAMES =

caseid

CoEx1_1 CoEx1_2 CoEx1_3 CoEx1_4 CoEx1_5 CoEx1_6 CoEx1_7 CoEx1_8

CoEx2_1 CoEx2_2 CoEx2_3 CoEx2_4 CoEx2_5 CoEx2_6

PPO_1 PPO_2 PPO_3 PPO_4 PPO_5 PPO_6

PNO_1 PNO_2 PNO_3 PNO_4 PNO_5 PNO_6

ETH_1 ETH_2 ETH_3 ETH_4 ETH_5 ETH_6;

USEVARIABLES =

CoEx1_1 CoEx1_2 CoEx1_3 CoEx1_4 CoEx1_5 CoEx1_6 CoEx1_7 CoEx1_8

CoEx2_1 CoEx2_2 CoEx2_3 CoEx2_4 CoEx2_5 CoEx2_6;

MISSING = All (-99);

MODEL:

CoEx_Pri BY

CoEx1_1 CoEx1_2 CoEx1_3 CoEx1_4 CoEx1_5 CoEx1_6 CoEx1_7 CoEx1_8;

CoEx_Pub BY

CoEx2_1 CoEx2_2 CoEx2_3 CoEx2_4 CoEx2_5 CoEx2_6;

CoEx BY CoEx_Pri CoEx_Pub;

CoEx_Pri@1;

CoEx_Pub@1;

CoEx@1;

!Modifications

COEX1_8 WITH COEX1_7;

COEX1_6 WITH COEX1_5;

COEX1_5 WITH COEX1_4;

COEX1_6 WITH COEX1_4;

COEX1_3 WITH COEX1_1;

OUTPUT:

STDYX MODINDICES (10) sampstat;

Multi-factor model of willingness to coexist

DATA: FILE = CoEx_Wolf.csv;

VARIABLE:

NAMES =

caseid

CoEx1_1 CoEx1_2 CoEx1_3 CoEx1_4 CoEx1_5 CoEx1_6 CoEx1_7 CoEx1_8

CoEx2_1 CoEx2_2 CoEx2_3 CoEx2_4 CoEx2_5 CoEx2_6

PPO_1 PPO_2 PPO_3 PPO_4 PPO_5 PPO_6

PNO_1 PNO_2 PNO_3 PNO_4 PNO_5 PNO_6

ETH_1 ETH_2 ETH_3 ETH_4 ETH_5 ETH_6;

USEVARIABLES =

CoEx1_1 CoEx1_2 CoEx1_3 CoEx1_4 CoEx1_5 CoEx1_6 CoEx1_7 CoEx1_8

CoEx2_1 CoEx2_2 CoEx2_3 CoEx2_4 CoEx2_5 CoEx2_6;

MISSING = All (-99);

MODEL:

CoEx_Pri BY

CoEx1_1 CoEx1_2 CoEx1_3 CoEx1_4 CoEx1_5 CoEx1_6 CoEx1_7 CoEx1_8;

CoEx_Pub BY

CoEx2_1 CoEx2_2 CoEx2_3 CoEx2_4 CoEx2_5 CoEx2_6;

!Modifications

COEX1_8 WITH COEX1_7;

COEX1_6 WITH COEX1_5;

COEX1_5 WITH COEX1_4;

COEX1_6 WITH COEX1_4;

COEX1_3 WITH COEX1_1;

OUTPUT:

STDYX MODINDICES (10) sampstat;

Single-factor model of willingness to coexist

DATA: FILE = CoEx_Wolf.csv;

VARIABLE:

NAMES =

caseid

CoEx1_1 CoEx1_2 CoEx1_3 CoEx1_4 CoEx1_5 CoEx1_6 CoEx1_7 CoEx1_8

CoEx2_1 CoEx2_2 CoEx2_3 CoEx2_4 CoEx2_5 CoEx2_6

PPO_1 PPO_2 PPO_3 PPO_4 PPO_5 PPO_6

PNO_1 PNO_2 PNO_3 PNO_4 PNO_5 PNO_6

ETH_1 ETH_2 ETH_3 ETH_4 ETH_5 ETH_6;

USEVARIABLES =

CoEx1_1 CoEx1_2 CoEx1_3 CoEx1_4 CoEx1_5 CoEx1_6 CoEx1_7 CoEx1_8

CoEx2_1 CoEx2_2 CoEx2_3 CoEx2_4 CoEx2_5 CoEx2_6;

MISSING = All (-99);

MODEL:

CoEx BY

CoEx1_1 CoEx1_2 CoEx1_3 CoEx1_4 CoEx1_5 CoEx1_6 CoEx1_7 CoEx1_8

CoEx2_1 CoEx2_2 CoEx2_3 CoEx2_4 CoEx2_5 CoEx2_6;

!Modifications

COEX1_8 WITH COEX1_7;

COEX1_6 WITH COEX1_5;

COEX1_5 WITH COEX1_4;

COEX1_6 WITH COEX1_4;

COEX1_3 WITH COEX1_1;

OUTPUT:

STDYX MODINDICES (10) sampstat;

Second-order factor model of beliefs about coexistence

DATA: FILE = CoEx_Wolf.csv;

VARIABLE:

NAMES =

caseid

CoEx1_1 CoEx1_2 CoEx1_3 CoEx1_4 CoEx1_5 CoEx1_6 CoEx1_7 CoEx1_8

CoEx2_1 CoEx2_2 CoEx2_3 CoEx2_4 CoEx2_5 CoEx2_6

PPO_1 PPO_2 PPO_3 PPO_4 PPO_5 PPO_6

PNO_1 PNO_2 PNO_3 PNO_4 PNO_5 PNO_6

ETH_1 ETH_2 ETH_3 ETH_4 ETH_5 ETH_6;

USEVARIABLES =

PPO_1 PPO_2 PPO_3 PPO_4 PPO_5 PPO_6

PNO_1 PNO_2 PNO_3 PNO_4 PNO_5 PNO_6

ETH_1 ETH_2 ETH_3 ETH_4 ETH_5 ETH_6;

MISSING = All (-99);

MODEL:

PPO BY

PPO_1 PPO_2 PPO_3 PPO_4 PPO_5 PPO_6;

PNO BY

PNO_1 PNO_2 PNO_3 PNO_4 PNO_5 PNO_6;

ETH BY

ETH_1 ETH_2 ETH_3 ETH_4 ETH_5 ETH_6;

ATT BY PPO* PNO ETH;

ATT@1;

!Modifications

PPO_4	WITH PPO_1;

PPO_4	WITH PPO_3;

PPO_5	WITH PPO_2;

PNO_2	WITH PNO_1;

PNO_3	WITH PNO_1;

PNO_4	WITH PNO_3;

ETH_5	WITH ETH_4;

ETH_6	WITH ETH_4;

PPO_3	WITH PPO_1;

PNO_5	WITH PNO_2;

PNO_6	WITH PNO_5;

ETH_6	WITH ETH_3;

ETH_6	WITH ETH_5;

PNO_4	WITH PNO_1;

PNO_6	WITH PNO_2;

ETH_4	WITH ETH_3;

ETH_5	WITH ETH_3;

PNO_6	WITH PNO_1;

ETH_2	WITH ETH_1;

PNO_6	WITH PNO_3;

PPO_6	WITH PPO_4;

PPO_6	WITH PPO_1;

PPO_6	WITH PPO_3;

OUTPUT:

STDYX MODINDICES (10) sampstat;

Multi-factor model of beliefs about coexistence

DATA: FILE = CoEx_Wolf.csv;

VARIABLE:

NAMES =

caseid

CoEx1_1 CoEx1_2 CoEx1_3 CoEx1_4 CoEx1_5 CoEx1_6 CoEx1_7 CoEx1_8

CoEx2_1 CoEx2_2 CoEx2_3 CoEx2_4 CoEx2_5 CoEx2_6

PPO_1 PPO_2 PPO_3 PPO_4 PPO_5 PPO_6

PNO_1 PNO_2 PNO_3 PNO_4 PNO_5 PNO_6

ETH_1 ETH_2 ETH_3 ETH_4 ETH_5 ETH_6;

USEVARIABLES =

PPO_1 PPO_2 PPO_3 PPO_4 PPO_5 PPO_6

PNO_1 PNO_2 PNO_3 PNO_4 PNO_5 PNO_6

ETH_1 ETH_2 ETH_3 ETH_4 ETH_5 ETH_6;

MISSING = All (-99);

MODEL:

PPO BY

PPO_1 PPO_2 PPO_3 PPO_4 PPO_5 PPO_6;

PNO BY

PNO_1 PNO_2 PNO_3 PNO_4 PNO_5 PNO_6;

ETH BY

ETH_1 ETH_2 ETH_3 ETH_4 ETH_5 ETH_6;

!Modifications

PPO_4	WITH PPO_1;

PPO_4	WITH PPO_3;

PPO_5	WITH PPO_2;

PNO_2	WITH PNO_1;

PNO_3	WITH PNO_1;

PNO_4	WITH PNO_3;

ETH_5	WITH ETH_4;

ETH_6	WITH ETH_4;

PPO_3	WITH PPO_1;

PNO_5	WITH PNO_2;

PNO_6	WITH PNO_5;

ETH_6	WITH ETH_3;

ETH_6	WITH ETH_5;

PNO_4	WITH PNO_1;

PNO_6	WITH PNO_2;

ETH_4	WITH ETH_3;

ETH_5	WITH ETH_3;

PNO_6	WITH PNO_1;

ETH_2	WITH ETH_1;

PNO_6	WITH PNO_3;

PPO_6	WITH PPO_4;

PPO_6	WITH PPO_1;

PPO_6	WITH PPO_3;

OUTPUT:

STDYX MODINDICES (10) sampstat;

Single-factor model of beliefs about coexistence

DATA: FILE = CoEx_Wolf.csv;

VARIABLE:

NAMES =

caseid

CoEx1_1 CoEx1_2 CoEx1_3 CoEx1_4 CoEx1_5 CoEx1_6 CoEx1_7 CoEx1_8

CoEx2_1 CoEx2_2 CoEx2_3 CoEx2_4 CoEx2_5 CoEx2_6

PPO_1 PPO_2 PPO_3 PPO_4 PPO_5 PPO_6

PNO_1 PNO_2 PNO_3 PNO_4 PNO_5 PNO_6

ETH_1 ETH_2 ETH_3 ETH_4 ETH_5 ETH_6;

USEVARIABLES =

PPO_1 PPO_2 PPO_3 PPO_4 PPO_5 PPO_6

PNO_1 PNO_2 PNO_3 PNO_4 PNO_5 PNO_6

ETH_1 ETH_2 ETH_3 ETH_4 ETH_5 ETH_6;

MISSING = All (-99);

MODEL:

BLF BY

PPO_1 PPO_2 PPO_3 PPO_4 PPO_5 PPO_6

PNO_1 PNO_2 PNO_3 PNO_4 PNO_5 PNO_6

ETH_1 ETH_2 ETH_3 ETH_4 ETH_5 ETH_6;

!Modifications

PPO_4	WITH PPO_1;

PPO_4	WITH PPO_3;

PPO_5	WITH PPO_2;

PNO_2	WITH PNO_1;

PNO_3	WITH PNO_1;

PNO_4	WITH PNO_3;

ETH_5	WITH ETH_4;

ETH_6	WITH ETH_4;

PPO_3	WITH PPO_1;

PNO_5	WITH PNO_2;

PNO_6	WITH PNO_5;

ETH_6	WITH ETH_3;

ETH_6	WITH ETH_5;

PNO_4	WITH PNO_1;

PNO_6	WITH PNO_2;

ETH_4	WITH ETH_3;

ETH_5	WITH ETH_3;

PNO_6	WITH PNO_1;

ETH_2	WITH ETH_1;

PNO_6	WITH PNO_3;

PPO_6	WITH PPO_4;

PPO_6	WITH PPO_1;

PPO_6	WITH PPO_3;

OUTPUT:

STDYX MODINDICES (10) sampstat;

### Measurement Invariance

Willingness to coexist model

DATA: FILE = CoEx_Wolf.csv;

VARIABLE:

NAMES =

caseid

CoEx1_1 CoEx1_2 CoEx1_3 CoEx1_4 CoEx1_5 CoEx1_6 CoEx1_7 CoEx1_8

CoEx2_1 CoEx2_2 CoEx2_3 CoEx2_4 CoEx2_5 CoEx2_6

PPO_1 PPO_2 PPO_3 PPO_4 PPO_5 PPO_6

PNO_1 PNO_2 PNO_3 PNO_4 PNO_5 PNO_6

ETH_1 ETH_2 ETH_3 ETH_4 ETH_5 ETH_6;

USEVARIABLES =

CoEx1_1 CoEx1_2 CoEx1_3 CoEx1_4 CoEx1_5 CoEx1_6 CoEx1_7 CoEx1_8

CoEx2_1 CoEx2_2 CoEx2_3 CoEx2_4 CoEx2_5 CoEx2_6;

MISSING = All (-99);

ANYLYSIS: MODEL IS CONFIGURAL METRIC SCALAR;

MODEL:

CoEx_Pri BY

CoEx1_1 CoEx1_2 CoEx1_3 CoEx1_4 CoEx1_5 CoEx1_6 CoEx1_7 CoEx1_8;

CoEx_Pub BY

CoEx2_1 CoEx2_2 CoEx2_3 CoEx2_4 CoEx2_5 CoEx2_6;

!Modifications

COEX1_8 WITH COEX1_7;

COEX1_6 WITH COEX1_5;

COEX1_5 WITH COEX1_4;

COEX1_6 WITH COEX1_4;

COEX1_3 WITH COEX1_1;

OUTPUT:

STDYX MODINDICES (10) sampstat;

Beliefs about coexistence model

DATA: FILE = CoEx_Wolf.csv;

VARIABLE:

NAMES =

caseid

CoEx1_1 CoEx1_2 CoEx1_3 CoEx1_4 CoEx1_5 CoEx1_6 CoEx1_7 CoEx1_8

CoEx2_1 CoEx2_2 CoEx2_3 CoEx2_4 CoEx2_5 CoEx2_6

PPO_1 PPO_2 PPO_3 PPO_4 PPO_5 PPO_6

PNO_1 PNO_2 PNO_3 PNO_4 PNO_5 PNO_6

ETH_1 ETH_2 ETH_3 ETH_4 ETH_5 ETH_6;

USEVARIABLES =

PPO_1 PPO_2 PPO_3 PPO_4 PPO_5 PPO_6

PNO_1 PNO_2 PNO_3 PNO_4 PNO_5 PNO_6

ETH_1 ETH_2 ETH_3 ETH_4 ETH_5 ETH_6;

MISSING = All (-99);

ANYLYSIS: MODEL IS CONFIGURAL METRIC SCALAR;

MODEL:

PPO BY

PPO_1 PPO_2 PPO_3 PPO_4 PPO_5 PPO_6;

PNO BY

PNO_1 PNO_2 PNO_3 PNO_4 PNO_5 PNO_6;

ETH BY

ETH_1 ETH_2 ETH_3 ETH_4 ETH_5 ETH_6;

!Modifications

PPO_4	WITH PPO_1;

PPO_4	WITH PPO_3;

PPO_5	WITH PPO_2;

PNO_2	WITH PNO_1;

PNO_3	WITH PNO_1;

PNO_4	WITH PNO_3;

ETH_5	WITH ETH_4;

ETH_6	WITH ETH_4;

PPO_3	WITH PPO_1;

PNO_5	WITH PNO_2;

PNO_6	WITH PNO_5;

ETH_6	WITH ETH_3;

ETH_6	WITH ETH_5;

PNO_4	WITH PNO_1;

PNO_6	WITH PNO_2;

ETH_4	WITH ETH_3;

ETH_5	WITH ETH_3;

PNO_6	WITH PNO_1;

ETH_2	WITH ETH_1;

PNO_6	WITH PNO_3;

PPO_6	WITH PPO_4;

PPO_6	WITH PPO_1;

PPO_6	WITH PPO_3;

OUTPUT:

STDYX MODINDICES (10) sampstat;

### Discriminant Validity (Chi-Square Difference Testing)

DATA: FILE = CoEx_Wolf.csv;

VARIABLE:

NAMES =

caseid

CoEx1_1 CoEx1_2 CoEx1_3 CoEx1_4 CoEx1_5 CoEx1_6 CoEx1_7 CoEx1_8

CoEx2_1 CoEx2_2 CoEx2_3 CoEx2_4 CoEx2_5 CoEx2_6

PPO_1 PPO_2 PPO_3 PPO_4 PPO_5 PPO_6

PNO_1 PNO_2 PNO_3 PNO_4 PNO_5 PNO_6

ETH_1 ETH_2 ETH_3 ETH_4 ETH_5 ETH_6;

USEVARIABLES =

CoEx1_1 CoEx1_2 CoEx1_3 CoEx1_4 CoEx1_5 CoEx1_6 CoEx1_7 CoEx1_8

CoEx2_1 CoEx2_2 CoEx2_3 CoEx2_4 CoEx2_5 CoEx2_6;

MISSING = All (-99);

MODEL:

CoEx_Pri BY

CoEx1_1* CoEx1_2 CoEx1_3 CoEx1_4 CoEx1_5 CoEx1_6 CoEx1_7 CoEx1_8;

CoEx_Pub BY

CoEx2_1* CoEx2_2 CoEx2_3 CoEx2_4 CoEx2_5 CoEx2_6;

CoEx_Pri@1;

CoEx_Pub@1;

CoEx_Pri WITH CoEx_Pub (a1);

!Modifications

COEX1_8 WITH COEX1_7;

COEX1_6 WITH COEX1_5;

COEX1_5 WITH COEX1_4;

COEX1_6 WITH COEX1_4;

COEX1_3 WITH COEX1_1;

MODEL TEST: a1 = 1;

OUTPUT:

STDYX MODINDICES (10) sampstat;

## References

Adams Knopff, A., Knopff, K. H., & St. Clair, C. C. (2016). Tolerance for cougars diminished by high perception of risk. *Ecology and Society*, *21*(4), Article 33. https://doi.org/10.5751/ES-08933-210433

Amit, R., & Jacobson, S. K. (2017). Understanding rancher coexistence with jaguars and pumas: a typology for conservation practice. *Biodiversity and Conservation*, *26*(6), 1353–1374. https://doi.org/10.1007/s10531-017-1304-1

Bhatia, S., Suryawanshi, K., Redpath, S. M., & Mishra, C. (2021). Understanding people’s responses toward predators in the Indian Himalaya. *Animal Conservation*, *24*(3), 424–431. https://doi.org/10.1111/acv.12647

Bright, A. D., & Manfredo, M. J. (1996). A conceptual model of attitudes toward natural resource issues: A case study of wolf reintroduction. *Human Dimensions of Wildlife*, *1*(1), 1–21. https://doi.org/10.1080/10871209609359048

Bruskotter, J. T., Singh, A., Fulton, D. C., & Slagle, K. (2015). Assessing Tolerance for Wildlife: Clarifying Relations Between Concepts and Measures. *Human Dimensions of Wildlife*, *20*(3), 255–270. https://doi.org/10.1080/10871209.2015.1016387

Carter, N. H., Riley, S. J., & Liu, J. (2012). Utility of a psychological framework for carnivore conservation. *Oryx*, *46*(4), 525–535. https://doi.org/10.1017/S0030605312000245

Crook, S. E. S. (2019). Attitudes and Risk Perception Toward the Mountain Lion Bordering the Santa Cruz Mountains, California. *Society & Animals*, *27*(4), 427–451. https://doi.org/10.1163/15685306-12341590

Davenport, M. A., Nielsen, C. K., & Mangun, J. C. (2010). Attitudes Toward Mountain Lion Management in the Midwest: Implications for a Potentially Recolonizing Large Predator. *Human Dimensions of Wildlife*, *15*(5), 373–388. https://doi.org/10.1080/10871209.2010.507564

Dechner, A. (2021). Emotions and the tolerance of large carnivores: pumas in a crop-based landscape in Brazil. *Environmental Conservation*, *48*(2), 93–99. https://doi.org/10.1017/S0376892920000533

Frank, B., Glikman, J. A [Jenny Anne], Sutherland, M., & Bath, A. J. (2016). Predictors of Extreme Negative Feelings Toward Coyote in Newfoundland. *Human Dimensions of Wildlife*, *21*(4), 297–310. https://doi.org/10.1080/10871209.2016.1151965

Fulton, D. C., Manfredo, M. J., & Lipscomb, J. (1996). Wildlife value orientations: A conceptual and measurement approach. *Human Dimensions of Wildlife*, *1*(2), 24–47. https://doi.org/10.1080/10871209609359060

Glikman, J. A [Jenny A.], Ciucci, P., Marino, A., Davis, E. O., Bath, A. J., & Boitani, L. (2019). Local attitudes toward Apennine brown bears: Insights for conservation issues. *Conservation Science and Practice*, *1*(5), Article e25. https://doi.org/10.1111/csp2.25

Glikman, J. A [Jenny Anne], Vaske, J. J., Bath, A. J., Ciucci, P., & Boitani, L. (2012). Residents’ support for wolf and bear conservation: the moderating influence of knowledge. *European Journal of Wildlife Research*, *58*(1), 295–302. https://doi.org/10.1007/s10344-011-0579-x

Heneghan, M. D., & Morse, W. (2018). Finding our bearings: Understanding public attitudes toward growing black bear populations in Alabama. *Human Dimensions of Wildlife*, *23*(1), 54–70. https://doi.org/10.1080/10871209.2017.1386248

Hiroyasu, E. H., Miljanich, C. P., & Anderson, S. E. (2019). Drivers of support: The case of species reintroductions with an ill-informed public. *Human Dimensions of Wildlife*, *24*(5), 401–417. https://doi.org/10.1080/10871209.2019.1622055

Hosaka, T., Sugimoto, K., & Numata, S. (2017). Childhood experience of nature influences the willingness to coexist with biodiversity in cities. *Palgrave Communications*, *3*(1). https://doi.org/10.1057/palcomms.2017.71

Inskip, C., Carter, N., Riley, S., Roberts, T., & MacMillan, D. (2016). Toward Human-Carnivore Coexistence: Understanding Tolerance for Tigers in Bangladesh. *PloS One*, *11*(1), e0145913. https://doi.org/10.1371/journal.pone.0145913

Johansson, M., Frank, J., Støen, O.‑G., & Flykt, A. (2017). An Evaluation of Information Meetings as a Tool for Addressing Fear of Large Carnivores. *Society & Natural Resources*, *30*(3), 281–298. https://doi.org/10.1080/08941920.2016.1239290

Johansson, M., Sandström, C., Pedersen, E., & Ericsson, G. (2016). Factors governing human fear of wolves: moderating effects of geographical location and standpoint on protected nature. *European Journal of Wildlife Research*, *62*(6), 749–760. https://doi.org/10.1007/s10344-016-1054-5

Landon, A. C., Jacobs, M. H., Miller, C. A., Vaske, J. J., & Williams, B. d. (2020). Cognitive and Affective Predictors of Illinois Residents’ Perceived Risks from Gray Wolves. *Society & Natural Resources*, *33*(5), 574–593. https://doi.org/10.1080/08941920.2019.1664680

Lute, M. L., & Carter, N. H. (2020). Are We Coexisting With Carnivores in the American West? *Frontiers in Ecology and Evolution*, *8*, Article 48. https://doi.org/10.3389/fevo.2020.00048

Lute, M. L., & Gore, M. L. (2019). Broadening the aperture on coexistence with wildlife through the lenses of identity, risk and morals. In B. Frank, J. A. Glikman, & S. Marchini (Eds.), *Conservation biology: Vol. 23. Human-wildlife interactions: Turning conflict into coexistence* (pp. 45–64). Cambridge University Press.

Lute, M. L., Navarrete, C. D., Nelson, M. P., & Gore, M. L. (2016). Moral dimensions of human-wildlife conflict. *Conservation Biology : The Journal of the Society for Conservation Biology*, *30*(6), 1200–1211. https://doi.org/10.1111/cobi.12731

Lute, M. L., Serenari, C., Drake, M. D., Peterson, M. N., Jensen, J., Belyea, C., Olfenbuttel, C., & White, M. (2020). Modeling urban socio-ecological drivers of human– carnivore coexistence. *Journal of Urban Ecology*, *6*(1), Article juaa022. https://doi.org/10.1093/jue/juaa022

Marchini, S., & Macdonald, D. W. (2012). Predicting ranchers’ intention to kill jaguars: Case studies in Amazonia and Pantanal. *Biological Conservation*, *147*(1), 213–221. https://doi.org/10.1016/j.biocon.2012.01.002

Mcgovern, E. B., & Kretser, H. E. (2015). Predicting support for recolonization of mountain lions (Puma concolor) in the Adirondack park. *Wildlife Society Bulletin*, *39*(3), 503–511. https://doi.org/10.1002/wsb.557

Morzillo, A. T., Mertig, A. G., Hollister, J. W., Garner, N., & Liu, J. (2010). Socioeconomic factors affecting local support for black bear recovery strategies. *Environmental Management*, *45*(6), 1299–1311. https://doi.org/10.1007/s00267-010-9485-3

Nesbitt, H. K., Metcalf, A. L., Lubeck, A. A., Metcalf, E. C., Beckman, C., Smith, A. P., & Cummins, T. M. (2021). Collective Factors Reinforce Individual Contributions to Human‐Wildlife Coexistence. *The Journal of Wildlife Management*, *85*(6), 1280–1295. https://doi.org/10.1002/jwmg.22061

Riley, S. J., & Decker, D. J. (2000). Risk perception as a factor in Wildlife Stakeholder Acceptance Capacity for cougars in montana. *Human Dimensions of Wildlife*, *5*(3), 50–62. https://doi.org/10.1080/10871200009359187

Serenari, C. (2021). Conservation reliance and its influence on support for carnivore recovery. *Conservation Science and Practice*, *3*(5). https://doi.org/10.1111/csp2.382

Slagle, K. M., Bruskotter, J. T., & Wilson, R. S. (2012). The Role of Affect in Public Support and Opposition to Wolf Management. *Human Dimensions of Wildlife*, *17*(1), 44–57. https://doi.org/10.1080/10871209.2012.633237

Sponarski, C. C., Miller, C. A., Vaske, J. J., & Spacapan, M. R. (2016). Modeling Perceived Risk from Coyotes Among Chicago Residents. *Human Dimensions of Wildlife*, *21*(6), 491–505. https://doi.org/10.1080/10871209.2016.1190989

Wuensch, K. L., Jenkins, K. W., & Poteat, G. M. (2002). Misanthropy, idealism and attitudes towards animals. *Anthrozoös*, *15*(2), 139–149. https://doi.org/10.2752/089279302786992621

Zajac, R. M., Bruskotter, J. T., Wilson, R. S., & Prange, S. (2012). Learning to live with black bears: A psychological model of acceptance. *The Journal of Wildlife Management*, *76*(7), 1331–1340. https://doi.org/10.1002/jwmg.398

Zinn, H. C., & Pierce, C. L. (2002). Values, Gender, and Concern about Potentially Dangerous Wildlife. *Environment and Behavior*, *34*(2), 239–256. https://doi.org/10.1177/0013916502034002005
